# Supplementary figures and images for: Evolution of SARS-CoV-2 in white-tailed deer in Pennsylvania 2021–2024
Source: PLoS Pathog. 2025 Jan 24;21(1):e1012883. doi: 10.1371/journal.ppat.1012883 (PMC11781694; doi:10.1371/journal.ppat.1012883)

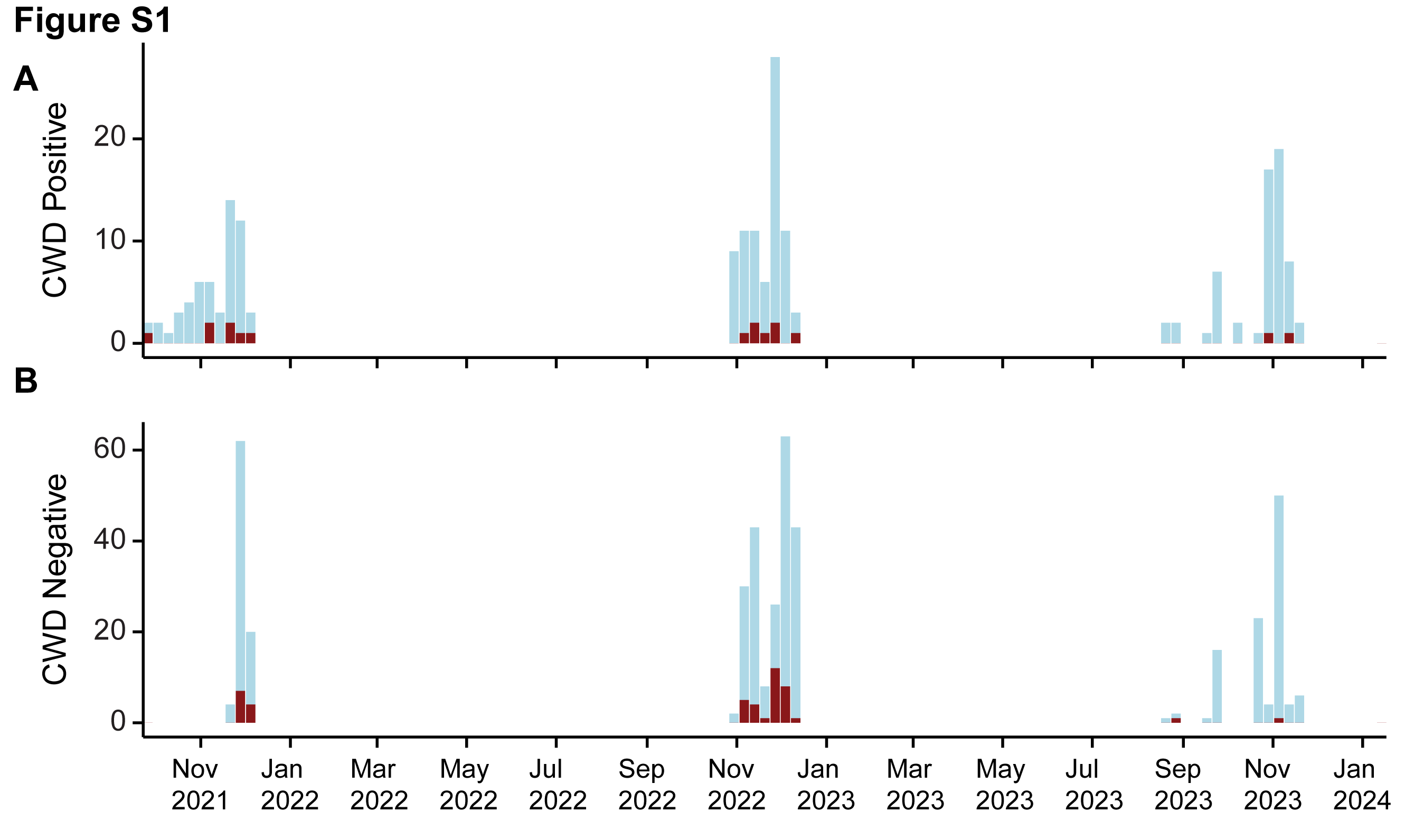

Supplement: S1 Fig — (A) CWD positive WTD are shown over the sampling period. Deer numbers are shown as stacked bar plots (y-axis) with blue indicating SARS-CoV-2 negative and red indicating SARS-CoV-2 positive by RT-qPCR. Time is shown on the x-axis. (B) CWD negative WTD sampled over time. Markings as in A with blue indicating SARS-CoV-2 negative and red indicating SARS-CoV-2 positive by RT-qPCR. The y-axis represents the counts. Red indicates SARS-CoV-2 positive and blue indicates SARS-CoV-2 negative by RT-qPCR. The x-axis labels apply to both panels. (TIF) [file ppat.1012883.s001.tif]

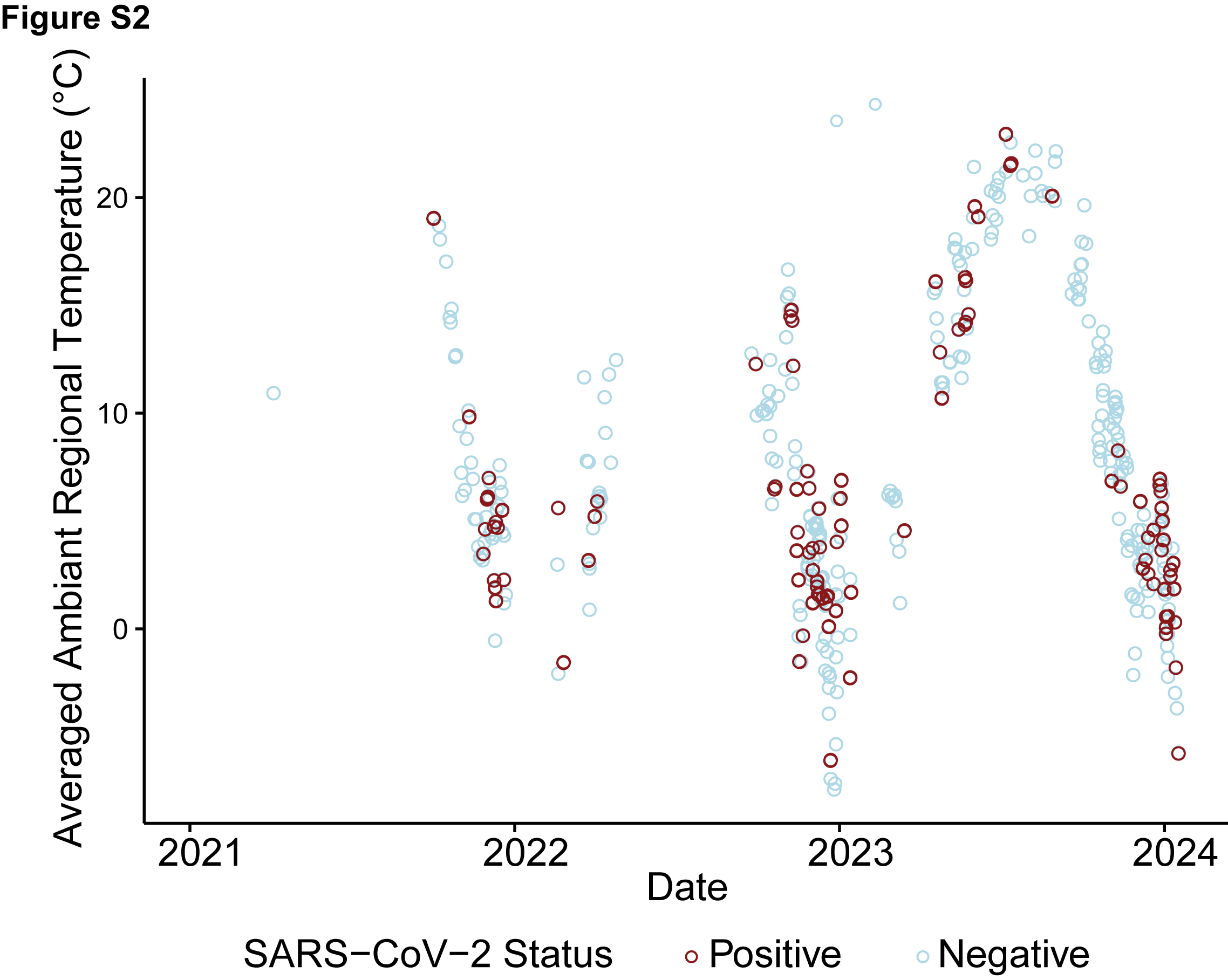

Supplement: S2 Fig — Each data point represents an individual WTD, colored by SARS-CoV-2 positivity, with blue indicating SARS-CoV-2 negative and red indicating SARS-CoV-2 positive by RT-qPCR. The y-axis represents temperature in Celsius averaged over the week of collection (x-axis) for the region that the sample was collected in. (TIF) [file ppat.1012883.s002.tif]

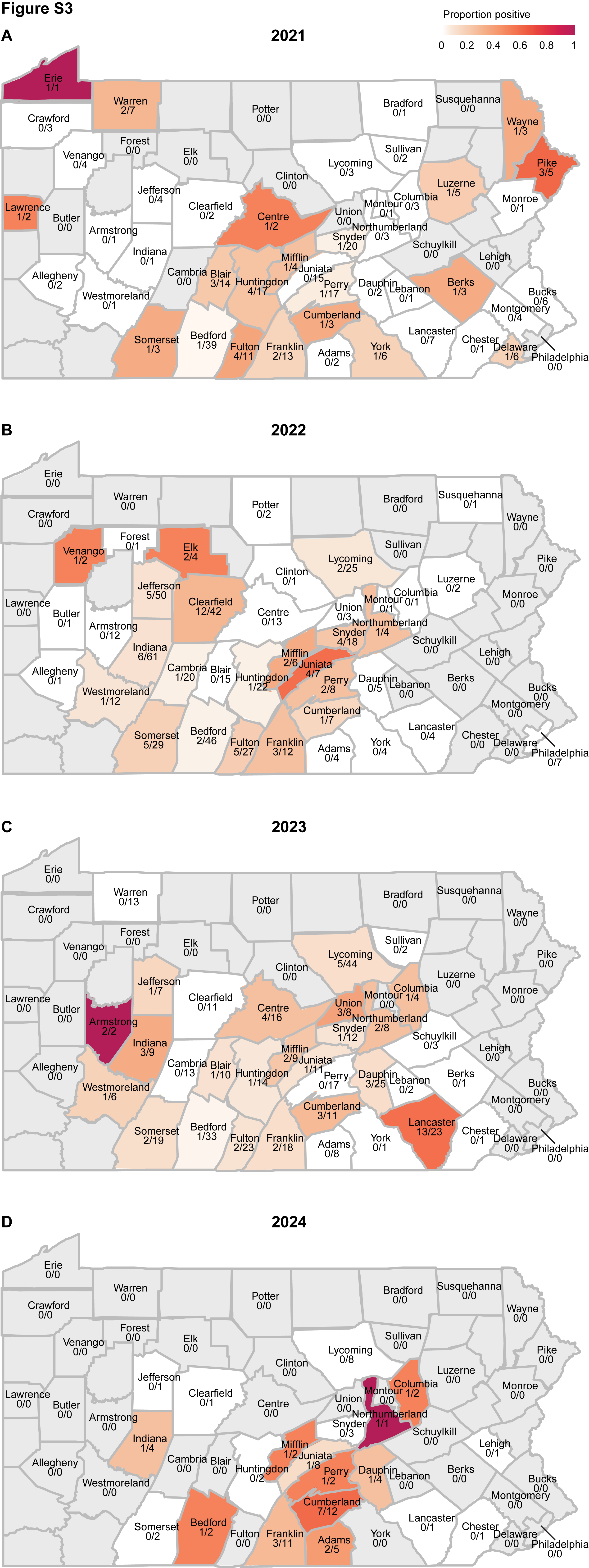

Supplement: S3 Fig — (A-D) Maps of Pennsylvania, with counties indicated. Each country is annotated for the ratio of positive SARS-CoV-2 WTD number of WTD tested. Grey indicates a county that had no tests performed that year. White indicates a county had tests performed and there were no positives. Shades of red indicate the proportion of SARS-CoV-2 positive specimens. Data are presented separately for (A) 2021 (B) 2022 (C) 2023, and (D) 2024. (TIF) [file ppat.1012883.s003.tif]

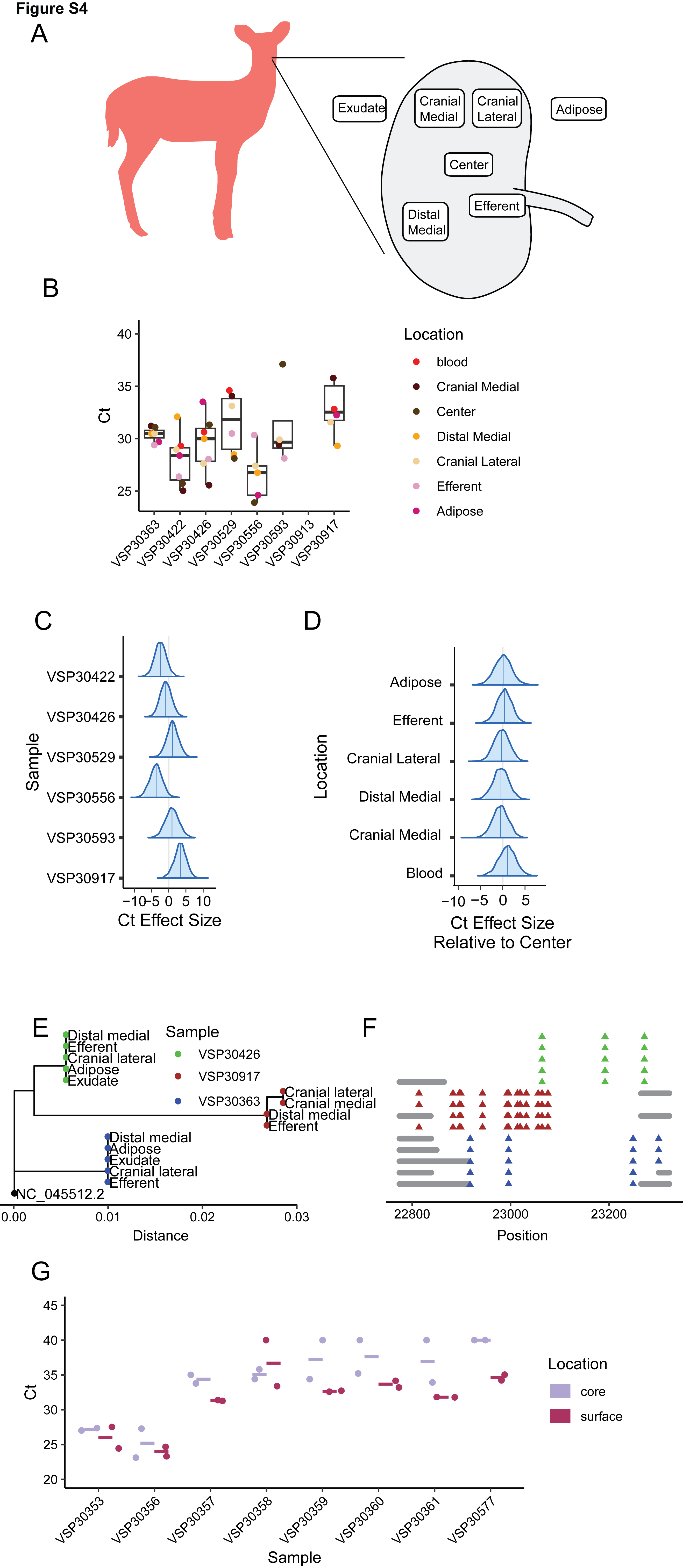

Supplement: S4 Fig — (A) Graphical illustration of WTD indicating RPLN locations of the seven sample types, including exudate, cranial medial, center, distal medial, cranial lateral, efferent, and adipose tissue. Seven WTD previously identified as SARS-CoV-2 positive were tested and one WTD previously identified as negative. (B) RT-qPCR Ct values stratified by sample and colored by RPLN site. Each dot represents the average of two replicates. Undetermined RT-qPCR results are indicated as points positioned at the top of the plot. “VSP” indicates “viral specimen number” (accession number). (C) Posterior distribution for Ct effect size of each sample (VSP) from a Bayesian linear mixed model. A value of 0 indicates that the variable had no effect on Ct value. (D) Posterior distribution for Ct effect size of each RPLN site. (E) Phylogenetic tree of spike-targeted sequencing results from three WTD, emphasizing within-animal consistency. (F) Paired with (E), map showing SNP location in the sequenced region of the SARS-CoV-2 spike, colored by sample, with grey indicating low coverage. (G) Ct value comparison of biopsied core vs superficial cuts across 8 WTD specimen with each dot representing a single replicate. The y-axis shows the Ct value; the x-axis shows the lymph node sampled. The bar represents an average across replicates. (TIF) [file ppat.1012883.s004.tif]

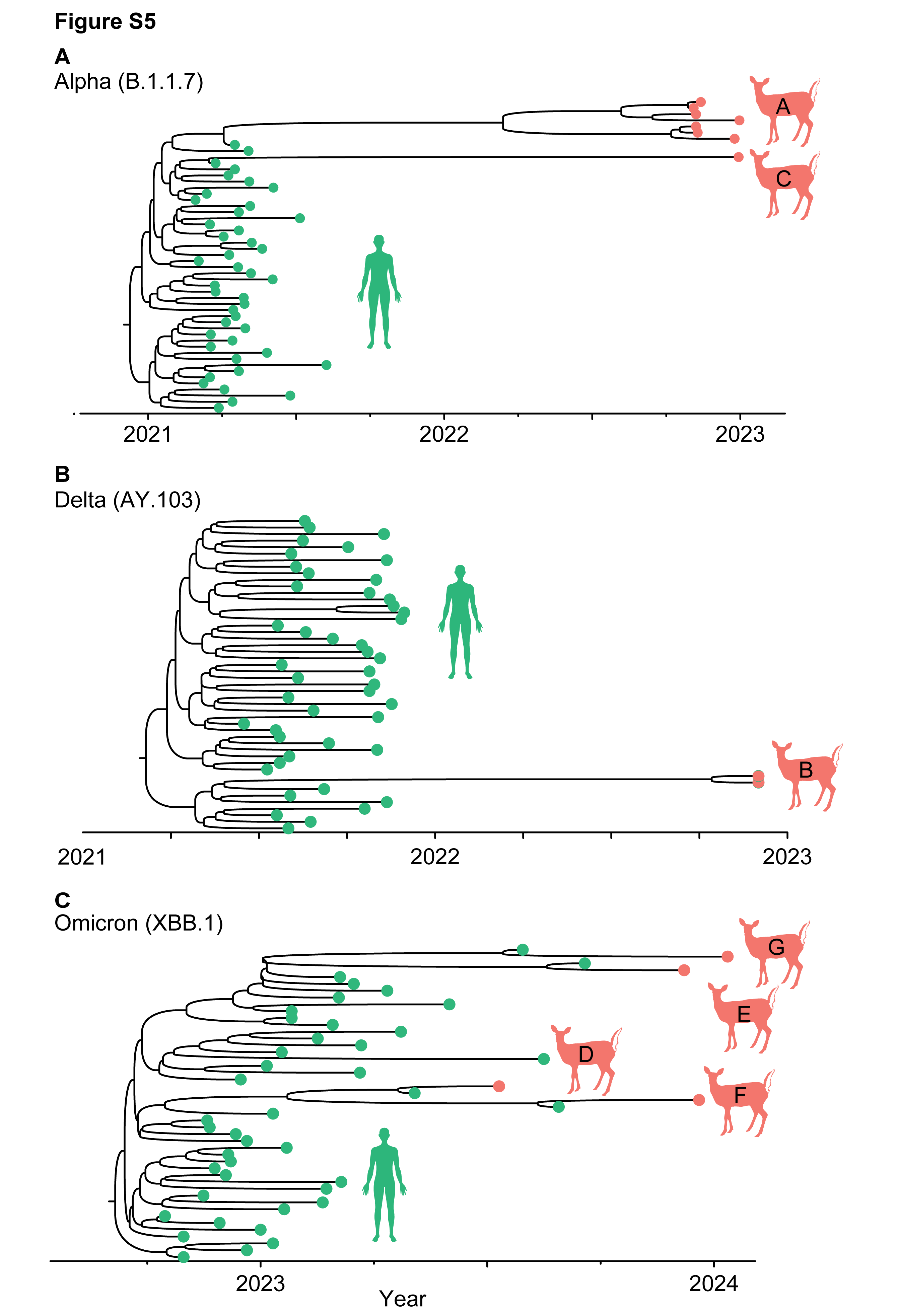

Supplement: S5 Fig — For all trees, colors reflect the host organism with green indicating human-derived isolates and pink indicating WTD-derived isolates. The icon of a WTD includes a letter specifying the cluster that the WTD belonged (Fig 5A-5G). (A) Time-resolved phylogeny of Alpha variant (B.1.1.7) WTD including nearest-neighbor sequences and subsampled background human sequences. (B) Time-resolved phylogeny of Delta variant (AY.103). (C) Time-resolved phylogeny of Omicron variant (XBB.1). (TIF) [file ppat.1012883.s005.tif]
