# Supplementary material for: Evolution of SARS-CoV-2 in white-tailed deer in Pennsylvania 2021–2024
Source: PLoS Pathog. 2025 Jan 24;21(1):e1012883. doi: 10.1371/journal.ppat.1012883 (PMC11781694; doi:10.1371/journal.ppat.1012883)
Supplement: Attachment — Submitted filename: 2024-08-12_PPATHOGENS-D-24-01513_SDI comments.pdf [file ppat.1012883.s009.pdf]

PLOS Pathogens
Evolution of SARS-CoV-2 in White-tailed Deer in Pennsylvania 2021-2024
--Manuscript Draft--

**Manuscript Number:**	PPATHOGENS-D-24-01513
**Full Title:**	Evolution of SARS-CoV-2 in White-tailed Deer in Pennsylvania 2021-2024
**Short Title:**	SARS-CoV-2 Persistence in White-tailed Deer
**Article Type:**	Research Article
**Section/Category:**	Evolutionary Biology - Virus
**Keywords:**	SARS-CoV-2; White-tailed deer; Virology; Evolution; Viral dynamics; Phylodynamics; Spillover; Zoonotic reservoir; Pennsylvania; Human-to-deer transmission; Chronic Wasting Disease (CWD); Retropharyngeal lymph nodes (RPLN); Bayesian logistic regression; Geographic distribution; Bayesian molecular clocks; Onward transmission
**Abstract:**	SARS-CoV-2 continues to transmit and evolve in humans and animals. White-tailed deer (Odocoileus virginianus) have been previously identified as a zoonotic reservoir for SARS-CoV-2 with high rates of infection and probable spillback into humans. Here we report sampling 1,127 white-tailed deer in Pennsylvania, and a genomic analysis of viral dynamics spanning 1,017 days between April 2021 and January 2024. To assess viral load and genotypes, RNA was isolated from retropharyngeal lymph nodes and analyzed using RT-qPCR and viral whole genome sequencing. Samples showed a 14.64% positivity rate by RT-qPCR. Analysis showed no association of SARS-CoV-2 prevalence with age, sex, or diagnosis with Chronic Wasting Disease. From the 165 SARS-CoV-2 positive deer, we recovered 25 whole genome sequences and an additional 17 spike-targeted amplicon sequences. The viral variants identified included 17 Alpha, 11 Delta, and 14 Omicron. Alpha largely stopped circulating in humans around September 2021, but persisted in deer as recently as March of 2023. Phylodynamic analysis of pooled genomic data from Pennsylvania documents at least 12 SARS-CoV-2 spillovers from humans into white-tailed deer, including a recent series of Omicron spillovers. Prevalence was higher in deer in regions with crop coverage rather than forest, suggesting an association with proximity to humans. Analysis of seasonality showed increased prevalence in winter and spring. These data document ongoing infections in white-tailed deer, probable onward transmission in deer, and a remarkable rate of new spillovers from humans.
**Additional Information:**	
**Question**	**Response**
**Government Employee**<br><br>Are you or any of the contributing authors an employee of the United States government?<br><br>Manuscripts authored by one or more US Government employees are not copyrighted, but are licensed under a CC0 Public Domain Dedication, which allows unlimited distribution and reuse of the article for any lawful purpose. This is a legal requirement for US Government employees.	No - No authors are employees of the U.S. government.

Powered by Editorial Manager® and ProduXion Manager® from Aries Systems Corporation

This will be typeset if the manuscript is accepted for publication.	
**Financial Disclosure**<br><br>Enter a financial disclosure statement that describes the sources of funding for the work included in this submission. Review the submission guidelines for detailed requirements. View published research articles from *PLOS Pathogens* for specific examples.<br><br>This statement is required for submission and **will appear in the published article** if the submission is accepted. Please make sure it is accurate.<br><br>**Funded studies**<br>Enter a statement with the following details:<br>• Initials of the authors who received each award<br>• Grant numbers awarded to each author<br>• The full name of each funder<br>• URL of each funder website<br>• Did the sponsors or funders play any role in the study design, data collection and analysis, decision to publish, or preparation of the manuscript?<br><br>Did you receive funding for this work?	The author(s) received no specific funding for this work.
**Competing Interests**<br><br>On behalf of all authors, disclose any competing interests that could be perceived to bias this work.<br><br>This statement will be typeset if the manuscript is accepted for publication.<br><br>Review the instructions link below and PLOS Pathogens' competing interests policy to determine what information must be disclosed at submission.	The authors have declared that no competing interests exist.
**Data Availability**	All relevant data are within the manuscript and its Supporting Information files.

Provide a **Data Availability Statement** in the box below. This statement should detail where the data used in this submission can be accessed. This statement will be typeset if the manuscript is accepted for publication.<br><br>Before publication, authors are required to make all data underlying their findings fully available, without restriction. Review our PLOS Data Policy page for detailed information on this policy. Instructions for writing your Data Availability statement can be accessed via the Instructions link below.	

Powered by Editorial Manager® and ProduXion Manager® from Aries Systems Corporation

Manuscript

Click here to access/download;Manuscript;marques_persistence-in-

Evolution of SARS-CoV-2 in White-tailed Deer in Pennsylvania 2021-2024

Andrew D. Marques[1], Matthew Hogenauer[1], Natalie Bauer[2], Michelle Gibison[2], Beatrice DeMarco[2], Scott Sherrill-Mix[3], Carter Merenstein[1], Ronald G. Collman[4], Roderick B. Gagne[2#], and Frederic D. Bushman[1#]

[1] Department of Microbiology, Perelman School of Medicine, University of Pennsylvania, Philadelphia, PA 19104

[2] Department of Pathobiology, Wildlife Futures Program, University of Pennsylvania School of Veterinary Medicine, New Bolton Center, Kennett Square, Pennsylvania, USA

[3] Department of Microbiology, Genetics, and Immunology, College of Veterinary Medicine, Michigan State University, East Lansing, Michigan 48824

[4] Division of Pulmonary, Allergy, and Critical Care, Philadelphia, Pennsylvania, 19104

[#]Corresponding author

Abstract

SARS-CoV-2 continues to transmit and evolve in humans and animals. White-tailed deer (*Odocoileus virginianus*) have been previously identified as a zoonotic reservoir for SARS-CoV-2 with high rates of infection and probable spillback into humans. Here we report sampling 1,127 white-tailed deer in Pennsylvania, and a genomic analysis of viral dynamics spanning 1,017 days between April 2021 and January 2024. To assess viral load and genotypes, RNA was isolated from retropharyngeal lymph nodes and analyzed using RT-qPCR and viral whole genome sequencing. Samples showed a 14.64% positivity rate by RT-qPCR. Analysis showed no association of SARS-CoV-2 prevalence with age, sex, or diagnosis with Chronic Wasting Disease. From the 165 SARS-CoV-2 positive deer, we recovered 25 whole genome sequences and an additional 17 spike-targeted amplicon sequences. The viral variants identified included 17 Alpha, 11 Delta, and 14 Omicron. Alpha largely stopped circulating in humans around September 2021, but persisted in deer as recently as March of 2023. Phylodynamic analysis of pooled genomic data from Pennsylvania documents at least 12 SARS-CoV-2 spillovers from humans into white-tailed deer, including a recent series of Omicron spillovers. Prevalence was higher in deer in regions with crop coverage rather than forest, suggesting an association with proximity to humans. Analysis of seasonality showed increased prevalence in winter and spring. These data document ongoing infections in white-tailed deer, probable

onward transmission in deer, and a remarkable rate of new spillovers from humans.

Introduction

COVID-19 is caused by severe acute respiratory syndrome coronavirus 2 (SARS-CoV-2). SARS-CoV-2 is highly transmissible among humans and has been responsible for more than 750 million reported cases and 7 million deaths globally since its emergence in late 2019[1]. In addition to humans, both wild and domestic animals have been reported to be susceptible. The virus likely arose in Rhinolophus bats[2-5], and recent studies document examples of infections in 55 vertebrate species[2,3,6-26] (Supp Table 1). For four of these species there are credible reports of spill-back into humans, including from wild white-tailed deer (WTD), domestic hamster, domestic cat, and farmed mink[14,27-29]. These findings focus intense interest on understanding potential variants that may emerge from SARS-CoV-2 infection in non-human vertebrates, and the consequences of those changes for human infection.

SARS-CoV-2 evolution often involves genetic jumps yielding variant viruses with more genetic changes per time than expected from continued human-to-human transmission. The origin of these new variants is not well understood. One origin may be prolonged infection of immunocompromised patients; another possibility is viral divergence in an animal species followed by spillback into humans[27,30-33]. Data in favor of the first theory include the documented emergence of drug-resistant substitutions and substitutions known to promote increased viral replication and immune evasion in immunocompromised patients with prolonged infections[32,34-38]. Several groups have reported measuring increased rates for within-host evolution in

immunocompromised subjects[31,34,35,39-41]. However, possible additional contributions of viral evolution and diversification in animal reservoirs are supported the by emergence of SARS in 2003 and MERS in 2012 from bats and camels[42,43].

Among possible sources of spillback from animals, WTD remain one of the most important species to examine because of the high infection rates and abundant populations in close proximity to humans. It is estimated that there are more than one million WTD in Pennsylvania alone[44,45]. Since 2020, positivity rates have been reported as high as 35.8% in some parts of the United States[46]. We and others have previously reported that specific SARS-CoV-2 variants persisted in WTD populations for months after circulation in humans had ceased[47,48]. Several studies suggest that lineages specific to WTD subsequently appeared in isolated human cases, suggesting spill back from WTD[27,48]. Thus it is critical to monitor the large WTD viral reservoir for evolution of new variants and possible onward transmission of evolved variants to humans.

Chronic wasting disease (CWD) is a prion-associated disease that may be a risk factor for SARS-CoV-2 in deer. CWD is a transmissible spongiform encephalopathy that affects the nervous system, takes about 16 months from exposure to symptom onset, and is invariably fatal to deer. CWD-positive deer show progressive weight loss, excessive urination, excessive salivation, and behavioral changes including stumbling and loss of fear of humans. To date, little is known about the possible risk factors associated with CWD and SARS-CoV-2,

and whether the prion disease could contribute to the rise of new SARS-CoV-2 variants.

Here, we examine SARS-CoV-2 in Pennsylvania from 2021 to 2024 over 1,127 newly collected WTD specimens. We investigated the dynamics of infection and the interplay between CWD and SARS-CoV-2.

Results

<u>SARS-CoV-2 Prevalence in WTD in Pennsylvania</u>

We sampled 1,127 WTD retropharyngeal lymph nodes (RPLNs) collected from deer between 4/5/2021 and 1/17/2024 from 39 counties in Pennsylvania (Supp Table 2). These data complement and extend our previous study (n=123 WTD samples)[47]. RPLNs are one of many sites known to harbor SARS-CoV-2 RNA in infected animals[49], and are a sample of convenience because RPLNs are among the tissues collected in the course of monitoring for CWD[49]. Cause of death for sampled animals included hunting (n = 414), roadkill (n = 731), and targeted removal (n = 12). Positivity was assessed through reverse-transcription quantitative polymerase chain reaction (RT-qPCR) using two different primers targeting distinct regions within the SARS-CoV-2 nucleocapsid[50]. We identified a total of 165/1,127 RPLN samples that were SARS-CoV-2 positive by RT-qPCR, for a total of 14.64% positive (95% confidence interval (CI) 12.7-16.9%).

Fig 1. RT-qPCR results for pooled Pennsylvania WTD samples from 2021-2024. In (A) counts of WTD are stratified by age and colored by RT-qPCR results with

(B) Ct values of positive samples. In (C) RT-qPCR counts are stratified by cause of death with (D) the associated Ct values. In (E) RT-qPCR counts are stratified by sex with (F) the associated Ct values.

Age was not significantly associated with SARS-CoV-2 positivity when comparing across groups of fawns (age 0-12 months, n=128), yearlings (age 13-24 months, n = 549), and adults (age 25 months or older, n = 396) (Pearson's chi-squared p=0.92) (Fig 1A). Looking only at positive samples, age was also not significantly associated with differences in Ct values (ANOVA p=0.41) (Fig 1B).

Cause of death was significantly associated with SARS-CoV-2 positivity when comparing hunter harvested (n=340), roadkill (n=710), and targeted removal (n=12) (Pearson's chi-squared p < 0.0001) (Fig 1C). A post-hoc pairwise comparison of proportions adjusting for multiple comparisons using Bonferroni correction suggested that targeted removals had a significantly higher positivity rate than hunter harvested (p<0.0001), and roadkill (p<0.0001) WTD. Targeted removals represent only a small subset (n=12) of all WTD collected (n=1,127). Eleven WTD were removed from the same county on the same day, and 10 of these tested positive for SARS-CoV-2, indicating removal of an infected cluster of animals which drove the statistical significance. Causes of death showed no association with SARS-CoV-2 Ct values (ANOVA p=0.57) (Fig 1D).

The animal's sex was not significantly associated with SARS-CoV-2 positivity (males: n=490; females: n=587; 2-sample test for equality of

proportions with continuity correction p=0.4249, males = 65/490 and females = 89/587) (Fig 1E). Animal sex was also not significantly associated with average SARS-CoV-2 Ct (Welch 2-sample t-test p=0.1132) (Fig 1F). The mean Ct for males was 32 (standard deviation (sd)=3.94) and for females was 31 (sd=3.80).

CWD	Counts	Counts Positive	Percent Positive
Negative	408	44	10.78%
Positive	196	16	8.16%

Table 1. Pooled CWD-positive WTD tested for SARS-CoV-2 compared to contemporaneously collected CWD-negative WTD. CWD column indicates the groups that are CWD positive or CWD negative. The counts positive and percent positive columns indicate the counts and percent SARS-CoV-2 positive for each CWD group.

Fig S1. Comparison of CWD (A) and non-CWD (B) samples colored by SARS-CoV-2 positivity over the three years of collection 2021-2023.

CWD samples were collected primarily during the hunting season over the course of 3 years (2021: September-December; 2022: October-December; and 2023: August-November). When comparing CWD-negative samples to CWD-positive samples collected at the same times, there was no significant difference in SARS-CoV-2 positivity (Two-sample test for equality of proportions with continuity correction p=0.38) (Fig S1, Table 1). Among SARS-CoV-2 positive

samples, there was no significant relationship between CWD status and Ct value (Welch two-sample t-test p=0.18).

Seasonality of Pennsylvania WTD SARS-CoV-2 Positivity

Aggregating our RT-qPCR data from 2021 to 2024 (Figure 2), including samples from our previous publication[47] and those newly determined here, showed significant differences in SARS-CoV-2 positivity by season (Pearson's Chi-squared $p<0.0001$) comparing spring (n=124, March 19-June 20), summer (n=41, June 21-September 21), fall (n=706, September 22-December 20), and winter (n=209, December 21-March 18) (Fig 2A). We also observed a significant difference in average Ct value by season (ANOVA p=0.0323), where samples taken in winter had significantly higher viral RNA levels (lower Ct) than positive SARS-CoV-2 samples in the fall (Tukey multiple comparisons of means p=0.016) (Fig 2B).

Fig 2. RT-qPCR for Pennsylvania Pooled WTD 2021-2024 Seasonality Data. In (A) RT-qPCR counts stratified by season with (B) the associated Ct values. (C) Prevalence in aggregate data from RPLN and nasal-swabs binned by week. Light blue indicates SARS-CoV-2 negative and dark red indicates SARS-CoV-2 positive. (D) Auto-regressive moving average logistic regression. Grey indicates the 95% credible interval.

Fig S2. Bayesian model performance for estimating seasonal effect. (A) Posterior distribution model intercept with (B) multi-chain exploration of parameter space. (C) Posterior distribution of effect size of sex being male compared to female with (D) multi-chain exploration of parameter space. (E) Posterior distribution of effect size of collecting samples in spring compared to fall with (F) multi-chain exploration of parameter space. (G) Posterior distribution of effect size of collecting samples in summer compared to fall with (H) multi-chain exploration of parameter space. (I) Posterior distribution of effect size of collecting samples in winter compared to fall with (J) multi-chain exploration of parameter space.

Fig S3 RT-qPCR positivity over time and weekly temperature at time of collection. Each data point represents an individual animal, colored by SARS-CoV-2 positivity. Y axis represents temperature in Celsius averaged over the week of collection for the region of sampling.

To probe this further, we developed a Bayesian logistic regression model to determine the estimated effect sizes of multiple variables, including fixed variables (season, ambient temperature during collection, and sex), and a random effect for cause of death (Fig S2). We include animal sex in our model to examine the model's performance with a variable that we would not expect to have an effect. As expected, sex did not correlate with SARS-CoV-2 positivity (males showed a 0.96 difference in odds more likely to be positive than females, 95% credible interval (CrI) (0.66-1.36)). Average weekly temperature from the

time of sample collection (for the region in which the sample was collected) was used to determine whether ambient temperature might contribute to positivity rate, possibly by modulating sample degradation (e.g. samples collected during warmer weeks might have lower positivity rate due to increased RNA degradation) (Fig S3). Temperature had no predictive effect on positivity (odds ratio = 0.97, 95% CrI 0.93-1.01). Season had a significant correlation with SARS-CoV-2 positivity with winter having 2.25-fold increased odds (95% CrI 1.47-3.47) compared to fall, and spring having a 2.06-fold increased odds (95% CrI 1.21-3.50) compared to fall. Summer and fall did not show a credible difference for positivity rates from each other.

To model positivity over time in our data, we used a Bayesian autoregressive moving average logistic regression to smooth data and estimate SARS-CoV-2 positivity rates in Pennsylvania WTD (Fig 2C and 2D). We observed several relative maxima that correlate with higher prevalence in winter and spring compared to fall and summer. We estimate average weekly SARS-CoV-2 positivity rates in the fall to be 9.8% (95% CrI 5.4% - 15.7%), and summer 7.5% (95% CrI 2.0%-18.6%) compared to winter 21.9% (95% CrI 12.2% - 34.7%) and spring 17.5% (95% CrI 8.7% - 31.1%).

Geographic spread of SARS-CoV-2 in WTD 2021-2024

To estimate positivity rates across different land use types, we used publicly available GIS data to determine the most common land use for each 5km radius around the sampled deer location[51](Fig 3A and 3B). We found that deer in

predominantly forested areas (13.16% positive, n = 623) were significantly less likely to be positive than deer in crop and pasture areas (21.21% positive, n=132) (p = 0.024, Chi-Squared). This suggests that proximity to human activity is associated with increased SARS-CoV-2 prevalence in WTD. The degree of surrounding landscape fragmentation, as measured by Moran's I, was not significantly different between positive and negative deer (p > 0.48, Wilcox test).

Fig 3. Land use and clustering analysis. Maps of Pennsylvania colored by land use category with latitude and longitude of WTD collections. (A) Map showing locations of SARS-CoV-2 positive WTD. (B) Map showing locations of SARS-CoV-2 negative WTD. (C) Stacked bar plot showing the proportion SARS-CoV-2 positive WTD from predominantly crops/pasture compared to forested areas.

Fig S4. SARS-CoV-2 WTD positive-positive pairs observed values by geographic distance compared to random chance and 95% CI. The red line indicates observed values, the black line indicates random chance, and the dashed grey lines indicate 95% CI.

Fig S5. Pennsylvania WTD positivity by year 2021-2024. Map of Pennsylvania counties containing ratio of positive SARS-CoV-2 WTD to number of WTD tested, colored by raw proportion. Grey indicates a country had no tests performed that year, white indicates a country had tests and there were no

positives, and hues of red indicate the raw proportion of SARS-CoV-2 positive specimen for (A) 2021, (B) 2022, (C) 2023, and (D) 2024.

To determine the extent of clustering of SARS-CoV-2 infections in WTD, we calculated the join-count statistic at varying spatial scales. The statistic assesses the frequency of positive-positive pairs for the binned time periods. Using a permutation test to determine enrichment, we found no significant effects of clustering (Fig S4). No trend was found for positivity by county when stratified by year 2021-2024 (Fig S5).

Characterization of the geographical distribution of viral variants via viral spike gene sequencing

To characterize the viral variants in WTD, we extracted RNA from positive samples and performed viral-targeted sequencing. Initial amplicon sequencing was performed using a nested primer set targeting the SARS-CoV-2 spike receptor binding domain (RBD) coding sequence, as previously described[47,52]. Forty-two of the 165 RT-qPCR positive samples yielded spike RBD sequences. This allowed us to identify 17 Alpha, 11 Delta, and 14 Omicron WTD-derived isolates. We found Alpha and Delta variants more than a year after they were last reported as the major circulating variants in humans, extending previous findings of protracted circulation in WTD (Fig 4A-C)[47,53]. We found that Alpha variants were observed clustered in WTD from the eastern half of the state and Delta variants clustered in the western half of the state (Fig 4D).

Fig 4. Circulating SARS-CoV-2 lineages in Pennsylvania WTD. In (A) all previously published WTD-derived SARS-CoV-2 with linked county-level geographic information. In (B) WTD-derived SARS-CoV-2 sequences contributed from this paper (collection spanning 2021-2024). (C) Human-derived isolates subsamples to 10,000 Pennsylvania sequences from January 2020 to February 2024. (D) Map of Pennsylvania showing ratio of samples testing positive for SARS-CoV-2, colored by estimated posterior proportion positive, with boxed labels containing month of collection and colored by SARS-CoV-2 variant.

<u>Analysis of viral whole genome sequences</u>

To investigate the relationship of WTD SARS-CoV-2 variants to those in humans, we determined 25 whole genome sequences from the 42 samples studied as spike amplicons. BLAST was used to determine the 5 most similar (nearest neighbor) sequences from the 5,025,365 available sequences from the USA on GISAID downloaded 3/25/2024 (Supp Table 3). Augur was then used to subsample 50 human sequences from anywhere in the USA and 100 additional sequences from Pennsylvania and its neighboring states, New York, New Jersey, Maryland, Delaware, West Virginia, and Ohio. Subsampling was done within each lineage identified in our WTD sequence data. These pooled sequences were then aligned using NextClade and IQ-Tree was used to construct a maximum-likelihood phylogenetic tree.

In these data, we identified 7 likely spillover events from human-to-deer. We did not detect any close matches of human and deer-specific lineages that would suggest deer-to-human spillback. We then pooled our newly determined WTD samples with previously published data from Pennsylvania WTD with >98% coverage from GISAID, which documented at least 12 total spillovers from humans to WTD in Pennsylvania since the start of the epidemic. With out limited dataset, we estimate approximately 1 spillover event every 68 days (collection 10/22/2021 to 1/12/2024).

Fig 5 Phylodynamics of Pennsylvania WTD based on whole genome sequencing. (A) Phylogenetic tree showing subsampled human-derived isolates spanning the course of the SARS-CoV-2 pandemic, including the earliest detection in the United States. Genomes are labeled by lineage, and colored by host species. Green indicates human-derived isolates, light red indicates WTD-derived isolates new to this paper, and dark red indicates WTD-derived isolates from previous works. Labeled letters indicate the suspected seven independent spillover events documented in this work. (B) Mutation heatmap paired with the phylogenetic tree showing the location of SNPs throughout the SARS-CoV-2 genome, colored by host species. (C) Root-to-tip plot showing genetic divergence as the number of mutations from first introduction to the United States, colored by SARS-CoV-2 lineage with open circles indicatacting WTD-derived isolates and closed circles indicating human-derived isolates.

We identified Alpha and Delta variants persisting in WTD for months to years after they had largely ceased to circulate in humans. This suggests long-term onward spread of SARS-CoV-2 in WTD, establishing WTD as a reservoir for ancestral lineages. Moreover, the Alpha strains that persisted in deer were as divergent from its nearest human-derived viral sequence as Omicron was from Alpha. The least divergent WTD Alpha lineage was 72 mutations from its nearest human-derived sequence.

The most recent isolates from WTD were found to be exclusively Omicron, marking a shift in circulating lineages within Pennsylvania WTD. This recent increase in WTD positivity for winter 2024 shows what appear to be 4 independent XBB.1 spillovers from humans into deer, coinciding with an increase in human cases of this variant. However, the more recent JN.1 lineage of the Omicron variant, which is largely driving the recent spike of SARS-CoV-2 human cases, has not been identified in Pennsylvania WTD samples to date.

An analysis of evolution rates within variants suggested that there is no significant difference in rates between human-circulating variants compared to deer-circulating variants (p=0.94). We then asked if there were greater than expected mutations in deer isolates when accounting for substitutions that might emerge during the original introduction to deer from humans. To test this we used a permutation test to determine whether deer samples were more likely than randomly selected human samples to be outliers from the historic evolution rate. This test found a non-significant trend with a probability of 0.0523. Together, there is no evidence given this limited data that jumping from human-to-deer

resulted in a higher genetic divergence rate in WTD than that observed in humans. Further, once circulating in deer, the virus did not diverge at significantly faster rate than observed in human-to-human transmission.

Fig S6. Time resolved viral phylogenetic trees. (A) Time-resolved phylogeny of Alpha variant (B.1.1.7) WTD including nearest-neighbor sequences and subsampled background human sequences. (B) Time-resolved phylogeny of Delta variant (AY.103). (C) Time-resolved phylogeny of Omicron variant (XBB.1).

We performed time-resolved phylogenetic analyses using BEAST to estimate the time to the most recent common ancestor for our 7 spillover events (Fig S6). Alpha cluster A was estimated to have its last common shared human ancestor on 4/1/2021 (95% highest posterior density (HPD) 2/5/2021 to 4/15/2021). This would suggest persistence of this Alpha lineage within deer for 1.76 years (95% CrI 1.72-1.91 years) which is a minimum estimate as it assumes that the spillover occurred at the time of the last common shared ancestor and not before. The analysis also assumes that the spillover was directly from humans to deer without an intermediate host. Alpha cluster C was estimated to have its last common ancestor with humans on 3/14/2021 (95% HPD 2/15/2021 to 3/22/2021), suggesting potential circulation in deer for 1.81 years (95% CrI 1.78-1.88 years).

The Delta variant also appeared to persist in WTD. Cluster B was estimated to share its last common ancestor on 5/28/2021 (95% HPD 3/27/2021

to 8/15/2021), corresponding to 1.52 years (95% HPD 1.30-1.69 years) of persistence in deer.

The XBB.1 Omicron lineage had an estimated 4 independent spillover events with their most recent common ancestor dating to 4/17/2023 (95% HPD 3/14/2023 to 5/5/2023), 8/19/2023 (95% HPD 6/11/2023 to 9/19/2023), 8/7/2023 (95% HPD 6/22/2023 to 8/29/2023), and 7/15/2023 (95% HPD 5/24/2023 to 7/31/2023). This gives a minimum estimated persistence of 0.24 years (95% CrI 0.19-0.33 years), 0.30 years (95% CrI 0.22-0.49 years), 0.37 years (95% CrI 0.31-0.50 years), and 0.50 years (95% CrI 0.45-0.64 years).

Fig S7. dN/dS analysis. Heatmap for SARS-CoV-2 coding regions and WTD-derived isolates with labeled ratio for counts of nonsynonymous substitutions to synonymous substitutions colored by dN/dS ratio.

To assess which elements within the viral genome were under the most selective pressure, we performed a dN/dS analysis over SARS-CoV-2 coding regions for each WTD whole genome sequence recovered (Fig S7). We found differences were not statistically significant when comparing dN/dS values among deer (Kruskal-Wallis p=0.053). However, there was a significant difference among coding regions (Kruskal-Wallis p<0.0001). Non-structural protein coding regions were under the greatest diversifying selection, including nsp9, ORF6, and ORF7a. This contrasts with human-derived isolates where

structural components including spike, nucleocapsid, and envelope were among the genes under the strongest diversifying selective pressure[39,54-56].

Variation Within Lymph Nodes

To assess within-lymph node variability, we collected multiple samples from single lymph nodes and quantified Ct values. The seven locations sampled included 1) exudate from the area surrounding RPLN samples, 2) cranial medial portion of the RPLN, 3) center of the RPLN, 4) distal medial portion of the RPLN, 5) cranial lateral portion of the RPLN, 6) efferent vessel, and 7) adipose tissue removed from the surface of the RPLN (Fig 6A). We performed RT-qPCR across two replicates of each location for 8 WTD lymph nodes (Fig 6B). Seven of these were previously identified as SARS-CoV-2 positive and one as SARS-CoV-2 negative. We found a greater amount of Ct variation across RPLN locations compared to variation within WTD (standard deviation of WTD: 2.74, standard deviation of sampling RPLN location 3.32). To determine if there was a significant difference across RPLN locations, we performed a two-way ANOVA, and found no significant difference among sites ($p=0.93$). In contrast, there was a significant difference when comparing lymph node specimens from different WTD ($p=0.013$). We also performed a Bayesian regression analysis to estimate the effect size of samples and location on Ct values. Similarly, we found no significant effect from sampling location within the RPLN (Fig 6D) but a significant effect from deer-to-deer sampling (Fig 6C).

Fig 6. Consistency of SARS-CoV-2 sequences and viral RNA levels in different RPLN locations. (A) Graphical illustration of WTD with RPLN location and 7 sites for collecting trimmed tissues for RNA extraction. (B) RT-qPCR Ct values stratified by sample and colored by RPLN site. Each dot represents the average of two replicates. Undetermined RT-qPCR results are indicated as points positioned at the top of the plot. (C) Posterior distribution for Ct effect size of each sample from a Bayesian linear mixed model. (D) Posterior distribution for Ct effect size of each RPLN site. (E) Phylogenetic tree of spike-targeted sequencing results from three WTD. (F) Paired with (E), heatmap showing SNPs location in SARS-CoV-2 spike sequenced region, colored by sample, with grey indicating low coverage. (G) Comparison of biopsied core vs superficial cuts across 8 WTD specimen with each dot representing a single replicate.

To compare viral variants found in different RPLN locations, three samples including three different viral variants (VSP30363 (Delta), VSP30426 (Alpha), and VSP30917 (Omicron)) were analyzed by amplification and sequencing of spike RBD coding regions. Not all sites produced amplicons, but those that did clustered tightly with other sequences from the same samples (Fig 6E, 6F). Thus the lymph node sequences supported initial infection with a single viral lineage.

To test whether biopsy samples from the core of lymph nodes showed different results than samples from superficial tissue trimming, we collected 8 WTD samples known to be SARS-CoV-2 positive and performed RT-qPCR in replicates for the core versus surface cuts (Fig 6G). We found that biopsied core

samples have significantly higher Ct values than superficial cuts (p=0.0035, two-tailed paired t-test). We implemented a Bayesian multivariate regression analysis to control for variation across samples, which confirmed that cut depth had a significant role on Ct with surface cuts having a Ct of difference of -1.73 (95% CrI -0.80 to -2.65) compared to the biopsy cut.

Discussion

Here we report new geographic, temporal, and genomic data on the spread of SARS-CoV-2 in Pennsylvania WTD from 2021 to 2024. Our data provide evidence for seven further spillovers of SARS-CoV-2 from humans to deer in Pennsylvania; together with previous data, the results now support twelve independent spillovers to date. No clear examples were seen of spill back from WTD to humans in our data. Thus, spillover from human to deer appears to be frequent—how this is happening so efficiently remains unclear. We identified land use associated with crops and pasture as having a higher association with WTD prevalence than forest, suggesting increased prevalence associated with proximity to humans. Our data also identified a significant seasonal trend, with the highest infection rates in winter and spring. We found no differences in infection rates or viral RNA load associated with the prion disease CWD. Genomic analysis identified WTD as a reservoir for ancestral Alpha and Delta variants, which persisted in WTD for more than 1.5 years after transmission from humans.

Seasonality of SARS-CoV-2 in WTD may be attributed to multiple potential factors. It could be driven by 1) behavioral changes when deer aggregate in larger groups during the more resource-limited colder months, 2) aggregation associated with mating, or 3) environmental changes with colder-drier air allowing more efficient transmission of viral particles from deer-to-deer. Seasonality of other coronaviruses is well characterized in humans including coronaviruses 229E, OC43, and NL63[57]. Seasonal trends for SARS-CoV-2 in humans have previously been published, although it is still not clear how much of this is driven by climate-related changes, changes in host behavior, or the timing of emergence of new variants[58], adding interest to understanding WTD as a model. Interactions associated with breeding might also be seeding the seasonal outbreaks in deer. In Pennsylvania, WTD breed from mid-October to mid-December with a peak in mid-November[59]. Winter and early spring are also seasons when deer aggregate in larger groups, something that occurs less in the late spring as deer separate in part to avoid attracting predators[60]. These seasonal gatherings of larger groups could allow for more efficient deer-to-deer transmission.

Our genomic data support the long-term persistence and genetic divergence of SARS-CoV-2 Alpha and Delta in WTD more than a year after circulation ended for these variants in humans. Such long-term evolution has the potential to generate new variants insensitive to human immune responses against existing strains. The WTD reservoir could also sustain a viral lineage

outside of a largely immune/vaccinated population for long enough that human immunity wanes, enabling the re-emergence of ancestral lineages[61].

Analysis of co-infected WTD with SARS-CoV-2 and CWD has not been previously published, and here we report no association in the population sampled. The lack of association suggests that the behavioral changes, physiologic burden, or malnutrition associated with CWD does not influence animal transmission of SARS-CoV-2. However, information on disease progression in animal samples was not available. Thus whether there are differences between early CWD versus disease-progressed animals remains to be determined.

This study has several limitations. Insufficient genomic surveillance of circulating SARS-CoV-2 strains in humans more recently might cause us to miss examples of spill back from deer to humans. Genomic background sequences were selected following a subsampling scheme for most samples with the inclusion of most-similar publicly available sequences, so not all strains of possible relevance were compared. Sample size was a limitation in some of the statistical analysis. Samples were not available for deep immunological profiling.

In conclusion, these results document a highly active ongoing SARS-CoV-2 epidemic in WTD in Pennsylvania. This motivates extensive ongoing surveillance of WTD and other vertebrate species to quantify viral spread and monitor for possible animal-to-human spill back infections.

Methods

Sample Collection

Retropharyngeal lymph node (RPLN) samples from free-ranging white-tailed deer were submitted to the Wildlife Futures Program (WFP) at the Pennsylvania Animal Diagnostic Laboratory System – New Bolton Center (PADLS-NBC) for CWD diagnostic testing. Samples were primarily collected from hunter harvested deer and road killed deer. Fresh samples had various times from collection to receipt by the lab and may have been at ambient temperatures for several days but generally reached the lab within one week. Once received by the lab samples were stored at 4°C (1-5 days) until initial CWD testing. All samples were tested using BioRad's TeSeETM Short Assay Protocol (SAP) Combi Kit ELISA for CWD. RPLNs with confirmed negative or positive CWD status were then stored at –20°C until they were used in this study.

qPCR

RPLN tissue was trimmed for RT-qPCR analysis and placed in a Precellys 2 mL Hard Tissue Homogenizing Ceramic Bead Tube (REF: P000911-LYSK1-A) containing 500 µL of PBS. If needed the tube was inverted to ensure the sample was immersed in PBS prior to homogenization. Once the samples were added to their homogenization tubes, positive and negative extraction controls were generated. The negative extraction control was a blank containing only PBS, and the positive extraction control was PBS with cell-derived viral RNA doped in from an in-house RNA stock (gift from Dr. Susan Weiss, strain WA-1). After the controls were generated, all samples were homogenized. Crucially, the samples

had to be sufficiently homogenized to allow for testing, but not homogenized so thoroughly that they would obstruct the RNA extraction columns used in subsequent steps. Samples were homogenized at 18 Hz for 4 minutes in the Qiagen TissueLyser II.

RNA extraction for RT-qPCR was carried out using the Qiagen RNeasy Mini Kit (REF: 74106) as previously described[47,50]. Two regions of the viral nucleocapsid gene, nucleocapsid 1 (N1) and nucleocapsid 2 (N2), were targeted. CDC N1 Probe and Primers (REF: 10006713) were used for the detection of nucleocapsid 1, and CDC N2 Probe and Primers (REF: 10006713) were used to detect nucleocapsid 2. Both probe and primer sets were used with TaqMan Fast Virus 1-Step Mix (REF: 4444434). Each extracted RNA sample was tested for N1 and N2 in duplicate, and any sample where three of four RT-qPCR tests were positive was considered SARS-CoV-2 positive. A negative control from a blank RNA extraction, and a negative control from a no template qPCR were used. Two positive controls were generated by doping in synthetic RNA (one positive control) or cell culture-derived viral RNA (a second positive control) at the RNA extraction steps. The synthetic RNA was IDT SARS-CoV-2 Plasmid DNA (REF: 10006625) and the cell-derived viral RNA was from an in-house RNA stock (a gift of Weiss, strain WA-1).

Autoregressive Moving Average (positivity over continuous time)

To estimate smoothed positivity rates over time, we implemented a Bayesian autoregressive moving average model using Markov Chain Monte

Carlo sampling in STAN[62]. This estimates positivity rate for a given week that regresses to the average of the previous week and the rate of change from one week should be similar to rate of change from the previous week.

$$P_w = \frac{e^{X_w}}{\sum_{j=1}^{n} e^{X_{j,w}}}$$

$$X_w = X_{w-1} + change_w$$

$$change_w \sim Normal(change_{w-1}, \sigma)$$

Where P_w is the true positivity rate for week w. $change_w$ is the change in log odds for week w. The standard deviation, σ, had a prior gamma distribution with shape parameter of 1 and scale parameter of 2. X_w had the flat prior given by a normal distribution with mean of 0 and standard deviation of 10.

Logistic regression (positivity over multiple variables)

To estimate the effect of SARS-CoV-2 seasonality in WTD we used a Bayesian regression model for estimating the effect size of fixed effect variables for the season of infection, average weekly temperature of that region of Pennsylvania during time of sample collection, and animal sex. We define the region as the nearest NOAA weather observation station with average temperature recorded. We also included a random effect for cause of death. Parameters were estimated using Markov Chain Monte Carlo methods.

$$\text{logit}(P(Positivity = 1)) = \beta_0 + \beta_1 Sex + \beta_2 Season + \beta_3 Temperature + u_{Death_i}$$

Where *P(*Positivity = 1) represents the probability of a given sample testing SARS-CoV-2 positive, logit(*P*) is the log-odds of the probability *P*, β_0 represents the intercept, β_1 represents the effect for the season, β_2 represents the effect of male sex on SARS-CoV-2 positivity, β_3 represents the effect of average regional temperature during sample collection on SARS-CoV-2 positivity, and u_{Death_i} represents the random effect for the cause of death on animal's probability of SARS-CoV-2 positivity.

Spike-targeted Amplicon Sequencing

Amplicon sequencing targeted the spike coding region using nested PCR. All primer sequences are in Supplementary Table 2. Extracted RNA underwent reverse transcription and initial DNA amplification using the Superscript IV One-Step RT-PCR System. The reaction mixture consisted of 5 µL of extracted viral RNA, 0.25 µL of Superscript IV, 12.5 µL of 2X Platinum SuperFi RT-PCR mix, 1.25 µL of each specific primer at 10 µM concretation, and 4.75 µL of molecular grade water, totaling 25 µL. The cycling conditions were set to: 2 minutes at 25°C, 20 minutes at 50°C, 2 minutes at 95°C, followed by 25 cycles of 95°C for 2 minutes, 55°C for 30 seconds, and 70°C for 1 minute.

The second PCR, a nested reaction, added sequencing adapters. It included 5 µL of the product from the first reaction, 12.5 µL of 2X Q5 hot start master mix, 0.5 µL of dNTPs (10 mM), 1.25 µL of each primer (10 µM), and 7.5 µL of water, also totaling 25 µL. The conditions were 95°C for 2 minutes, followed

by 20 cycles of 95°C for 15 seconds, 55°C for 30 seconds, and 72°C for 1 minute.

The PCR product was visualized using a 1% agarose gel to determine whether any fragments of 550 base pairs were produced. Samples that had expected amplification band sizes were sent for Sanger sequencing at the UPENN sequencing core—no notable SARS-CoV-2 sequences were detected.

Whole Genome Sequencing

To sequence viral genomes, the POLAR protocol was used[63]. Initially, 5 µL of RNA sample was combined with several reagents: 0.5 µL of Random Hexamers, 0.5 µL of a dNTPs mix (10 mM), and 1 µL of nuclease-free water. This mixture was heated at 65°C for 5 minutes. For reverse transcription, 6.5 µL of this heated mixture was added to 0.5 µL of SuperScript III Reverse Transcriptase, 2 µL of First-Strand Buffer, 0.5 µL of DTT (0.1 mM), and 0.5 µL of RNaseOut, and then incubated at 42°C for 50 minutes followed by 10 minutes at 70°C.

For the amplification of cDNA, 2.5 µL of the transcription reaction was mixed with 0.25 µL of Q5 Hot Start DNA Polymerase, 5 µL of Q5 Reaction Buffer, 0.5 µL of a dNTPs mix (10 mM), and primers specific to the study (4.0 µL for the first set, 3.98 µL for the second set), with additional water to bring the total volume to 25 µL. The samples underwent 25 cycles of heating to 98°C for 30 seconds, cooling to 65°C for 5 minutes, then briefly heated again to 98°C.

Afterward, the two reactions were combined, and the DNA was prepared for sequencing using the Nextera XT Library Preparation Kit. Libraries were tagged using IDT DNA/RNA UD Indexes and quantified with the Quant-iT PicoGreen dsDNA assay. Libraries were sequenced on the Illumina MiniSeq platform.

Phylogenetic Analysis

Phylogenetic analysis was performed on samples downloaded from GISAID, subsampled using Augur v24.3.0, aligned using Nextclade v2.14.0, and maximum-likelihood trees inferred using IQ-TREE v1.6.12 with 1,000 bootstrap replicates. Our WTD-derived isolates were combined with 5 of the nearest-neighbor isolates, 100 isolates subsampled from Pennsylvania, New York, New Jersey, Delaware, West Virginia, Maryland, and Ohio and an additional 50 samples subsampled from anywhere within the USA (Supp Table 3). The 5 nearest-neighbor isolates were identified through a BLASTn query of all 5,025,365 isolates available on GISAID at the time of download (3/25/2024). The earliest detected isolate from a given lineage with complete date information and >99% coverage was included as the root. For the tree describing all lineages, the same protocol was followed above without restricting for lineage and all previously reported WTD and their 5 nearest neighbors that had >98% coverage were included. Trees were visualized using ggtree in the provided code.

Time-Resolved Phylogenetic Analysis

A time-scaled Bayesian maximum clade credibility (MCC) tree was used to estimate the introduction of SARS-CoV-2 lineages into PA white-tailed deer. We selected genomes based on their closest human neighbors using GISAID's database limiting results to isolates collected in the USA, resulting in a pool of 5,025,365 SARS-CoV-2 genomes from which the nearest 5 sequences were pulled using BLASTn. If a deer's closest matches included another deer, both were analyzed in the same tree. Sequences were filtered to have 98% coverage and exact collection dates were required. Subsampling was performed using Nextclade Augur tool on sequences downloaded from GISAID. We subsampled to include 100 human-derived isolates from the same lineage restricted to the sampled region (Pennsylvania, New York, New Jersey, Delaware, West Virginia, Maryland, and Ohio). The trees were rooted with the earliest detection of the lineage that had >99% coverage and complete collection data. An additional 50 sequences subsampled from anywhere in the USA was used. Each dataset was aligned using NextClade with Wuhan-Hu-1 as a reference (NC_045512.2). A Markov chain Monte Carlo (MCMC) method in BEAST v1.10.4 was used to generate Bayesian molecular clocks[64]. A general time reversible substitution model with gamma-distributed rate variation and an uncorrelated relaxed lognormal clock was implemented with a Bayesian Skyline tree with a group size of 10[46,47,65-67]. MCMC sampling ran for 100 million iterations, with every thousandth iteration sampled and 10 million iterations discarded as burn-in. BEAGLE 3 improved computational performance. Tracer v1.7.1 assessed convergence, TreeAnnotator v1.10.4 summarized the MCC tree, and FigTree

v1.4.4 visualized it[64,68]. All tree analyses were repeated three times independently to confirm convergence and effective sample sizes above 200. Maximum-likelihood trees were generated by IQ-TREE v1.6.12.

RPLN Subsampling Study

To examine the distribution of viral RNA levels and genomic diversity present within single RPLN samples, harvests from multiple locations on several specimen were conducted. Seven RPLNs were chosen for this experiment, six known RT-qPCT positives and one RT-qPCR negative included as a control. Sample locations included exudate and adipose tissue surrounding the RPLN and sections from cranial medial, distal medial, cranial lateral, efferent vessel, and center regions of the RPLN sample. For each sampling location, two pieces of tissue were harvested following the protocol above and processed independently. RT-qPCR and spike-targeted sequencing was performed as described above.

Geospatial analysis

Land use data were downloaded from the Pennsylvania Spatial Data Access (PASDA) portal (https://www.pasda.psu.edu/uci/DataSummary.aspx?dataset=2514). Land cover was determined from the 2021 Pennsylvania Cropland raster dataset at 30-meter resolution. All crops and pasture were collapsed into a single land use, as were all forests, all wetlands, and all developed land categories. The terra

(https://cran.r-project.org/web/packages/terra/index.html) and raster (https://cran.r-project.org/web/packages/raster/index.html) R packages were used for raster data processing, and vector data was processed with the sf package (https://cran.r-project.org/web/packages/sf/index.html). Join-count statistics were calculated manually in R.

Data availability

Viral sequences published in this study can be accessed through GISAID and NCBI using the accessions listed in Supp Table 2. Raw data can be accessed through SRA accession SUB14595397 and with BioProject accession PRJNA1134208. Previously published consensus sequences used in this work have accessions that can be found in Supp Table 3. Code can be accessed on GitHub at https://github.com/andrewdmarques/PA_WTD_Genomics. Key reagents are listed in Supp Table 4.

Ethics statement

This study did not involve live animals. Diagnostic samples from deceased animals were submitted to the Pennsylvania Animal Diagnostic Laboratory System at the New Bolton Center for CWD testing. The residual tissues were donated for this research.

No human participants were involved in the data collection for this study. Data referencing human-derived viral isolates were obtained from previously

published online databases. See the data availability section and supplemental table S3 for additional information.

Acknowledgements

We are grateful to members of the Bushman, Gagne and Collman laboratories for help and suggestions. We thank Laurie Zimmerman for help with artwork and manuscript preparation. We thank the numerous staff with the Pennsylvania Game Commission for the collection of the RPLN samples as well as Jan Yacabucci and John Armstrong for conducting the CWD diagnostic testing. This work was supported in part by the Penn University Research Foundation. Further funding was provided by a contract award from the Centers for Disease Control and Prevention (CDC BAA 200-2021-10986 and 75D30121C11102/000HCVL1-2021-55232), the Penn Center for Global Genomics and Health Equity Keystone Pilot Grant (GGHE-KP-2021-001), philanthropic donations to the Penn Center for Research on Coronaviruses and Other Emerging Pathogens, and by NIH grant R61/33-HL137063 and AI140442-supplement for SARS-CoV-2. Additional assistance was provided by the Penn Center for AIDS Research (P30-AI045008).

Supplementary Material

Supp Table 1. Animal species reported to be infectable with SARS-CoV-2. Only species with well documented infections from scientific articles (peer review or preprint) were consider credible reports of animal infection.

Supp Table 2. Sample metadata including WTD demographics, date of collection, cause of death, county of death, and sequence accession.

Supp Table 3. Samples and accession numbers for sequence data used in this study.

Supp Table 4. Key reagents.

References

1 Organization, W. H. COVID-19 cases | WHO COVID-19 dashboard. *datadot* (2024).

2 Meekins, D. A., Gaudreault, N. N. & Richt, J. A. Natural and Experimental SARS-CoV-2 Infection in Domestic and Wild Animals. *Viruses* **13**, doi:10.3390/v13101993 (2021).

3 Wacharapluesadee, S. *et al.* Evidence for SARS-CoV-2 related coronaviruses circulating in bats and pangolins in Southeast Asia. *Nat Commun* **12**, 972, doi:10.1038/s41467-021-21240-1 (2021).

4 Andersen, K. G., Rambaut, A., Lipkin, W. I., Holmes, E. C. & Garry, R. F. The proximal origin of SARS-CoV-2. *Nat Med* **26**, 450-452, doi:10.1038/s41591-020-0820-9 (2020).

5 Boni, M. F. *et al.* Evolutionary origins of the SARS-CoV-2 sarbecovirus lineage responsible for the COVID-19 pandemic. *Nat Microbiol* **5**, 1408-1417, doi:10.1038/s41564-020-0771-4 (2020).

6 Zhao, J. *et al.* Risk assessment of SARS-CoV-2 replicating and evolving in animals. *Trends Microbiol* **32**, 79-92, doi:10.1016/j.tim.2023.07.002 (2024).

7 Gomez, J. C. *et al.* Exposure to severe acute respiratory syndrome coronavirus 2 (SARS-CoV-2) in the endangered Iberian lynx (Lynx pardinus). *Vet Microbiol* **290**, 110001, doi:10.1016/j.vetmic.2024.110001 (2024).

8 Pereira, A. H. B. *et al.* A Novel Host of an Emerging Disease: SARS-CoV-2 Infection in a Giant Anteater (Myrmecophaga tridactyla) Kept Under Clinical Care in Brazil. *Ecohealth* **19**, 458-462, doi:10.1007/s10393-022-01623-6 (2022).

9 Vercammen, F. *et al.* SARS-CoV-2 Infection in Captive Hippos (Hippopotamus amphibius), Belgium. *Animals (Basel)* **13**, doi:10.3390/ani13020316 (2023).

10 Mahajan, S. *et al.* Detection of SARS-CoV-2 in a free ranging leopard (Panthera pardus fusca) in India. *Eur J Wildl Res* **68**, 59, doi:10.1007/s10344-022-01608-4 (2022).

11 Wang, L. *et al.* Detection of SARS-CoV-2 clade B.1.2 in three snow leopards. *Transbound Emerg Dis* **69**, e3346-e3351, doi:10.1111/tbed.14625 (2022).

12 Pereira, A. H. *et al.* Natural SARS-CoV-2 Infection in a Free-Ranging Black-Tailed Marmoset (Mico melanurus) from an Urban Area in Mid-West Brazil. *J Comp Pathol* **194**, 22-27, doi:10.1016/j.jcpa.2022.03.005 (2022).

13 Singh, D. K. *et al.* Responses to acute infection with SARS-CoV-2 in the lungs of rhesus macaques, baboons and marmosets. *Nat Microbiol* **6**, 73-86, doi:10.1038/s41564-020-00841-4 (2021).

14 Oude Munnink, B. B. *et al.* Transmission of SARS-CoV-2 on mink farms between humans and mink and back to humans. *Science* **371**, 172-177, doi:10.1126/science.abe5901 (2021).

15 Sit, T. H. C. *et al.* Infection of dogs with SARS-CoV-2. *Nature* **586**, 776-778, doi:10.1038/s41586-020-2334-5 (2020).

16 Chan, T. *et al.* Detection of SARS-CoV-2 RNA in a Zoo-Kept Red Fox (Vulpes vulpes). *Viruses* **16**, doi:10.3390/v16040521 (2024).

17 Takemura, T. *et al.* SARS-CoV-2 Infection in Beaver Farm, Mongolia, 2021. *Emerg Infect Dis* **30**, 391-394, doi:10.3201/eid3002.231318 (2024).

18 Tewari, D. *et al.* SARS-CoV-2 Infection Dynamics in the Pittsburgh Zoo Wild Felids with Two Viral Variants (Delta and Alpha) during the 2021-2022 Pandemic in the United States. *Animals (Basel)* **13**, doi:10.3390/ani13193094 (2023).

19 Wang, Y. *et al.* SARS-CoV-2 Exposure in Norway Rats (Rattus norvegicus) from New York City. *mBio* **14**, e0362122, doi:10.1128/mbio.03621-22 (2023).

20 Arteaga, F. L. *et al.* (bioRxiv, 2022).

21 Ulrich, L. *et al.* Experimental SARS-CoV-2 Infection of Bank Voles. *Emerg Infect Dis* **27**, 1193-1195, doi:10.3201/eid2704.204945 (2021).

22 Clancy, C. S. *et al.* Histologic pulmonary lesions of SARS-CoV-2 in 4 nonhuman primate species: An institutional comparative review. *Vet Pathol* **59**, 673-680, doi:10.1177/03009858211067468 (2022).

23 Goldberg, A. *et al.* (2022).

24 Melo, F. L. *et al.* (2022).

25 Lenz, O. C. *et al.* SARS-CoV-2 Delta Variant (AY.3) in the Feces of a Domestic Cat. *Viruses* **14**, doi:10.3390/v14020421 (2022).

26 Boggiatto, P. M. *et al.* Persistence of viral RNA in North American elk experimentally infected with an ancestral strain of severe acute respiratory syndrome coronavirus 2 (SARS-CoV-2). *Sci Rep* **14**, 11171, doi:10.1038/s41598-024-61414-7 (2024).

27 Pickering, B. *et al.* Divergent SARS-CoV-2 variant emerges in white-tailed deer with deer-to-human transmission. *Nat Microbiol* **7**, 2011-2024, doi:10.1038/s41564-022-01268-9 (2022).

28 Sila, T. *et al.* Suspected Cat-to-Human Transmission of SARS-CoV-2, Thailand, July-September 2021. *Emerg Infect Dis* **28**, 1485-1488, doi:10.3201/eid2807.212605 (2022).

29 Yen, H. L. *et al.* Transmission of SARS-CoV-2 delta variant (AY.127) from pet hamsters to humans, leading to onward human-to-human transmission: a case study. *Lancet* **399**, 1070-1078, doi:10.1016/S0140-6736(22)00326-9 (2022).

30 Markov, P. V. *et al.* The evolution of SARS-CoV-2. *Nat Rev Microbiol* **21**, 361-379, doi:10.1038/s41579-023-00878-2 (2023).

31 Borges, V. *et al.* Long-Term Evolution of SARS-CoV-2 in an Immunocompromised Patient with Non-Hodgkin Lymphoma. *mSphere* **6**, e0024421, doi:10.1128/mSphere.00244-21 (2021).

32 Gonzalez-Reiche, A. S. *et al.* Sequential intrahost evolution and onward transmission of SARS-CoV-2 variants. *Nat Commun* **14**, 3235, doi:10.1038/s41467-023-38867-x (2023).

33 Lu, L. *et al.* Adaptation, spread and transmission of SARS-CoV-2 in farmed minks and associated humans in the Netherlands. *Nat Commun* **12**, 6802, doi:10.1038/s41467-021-27096-9 (2021).

34 Leung, W. F. *et al.* COVID-19 in an immunocompromised host: persistent shedding of viable SARS-CoV-2 and emergence of multiple mutations: a case report. *Int J Infect Dis* **114**, 178-182, doi:10.1016/j.ijid.2021.10.045 (2022).

35 Truong, T. T. *et al.* Increased viral variants in children and young adults with impaired humoral immunity and persistent SARS-CoV-2 infection: A consecutive case series. *EBioMedicine* **67**, 103355, doi:10.1016/j.ebiom.2021.103355 (2021).

36 Monrad, I. *et al.* Persistent Severe Acute Respiratory Syndrome Coronavirus 2 Infection in Immunocompromised Host Displaying Treatment Induced Viral Evolution. *Open Forum Infect Dis* **8**, ofab295, doi:10.1093/ofid/ofab295 (2021).

37 Jensen, B. *et al.* Emergence of the E484K mutation in SARS-COV-2-infected immunocompromised patients treated with bamlanivimab in Germany. *Lancet Reg Health Eur* **8**, 100164, doi:10.1016/j.lanepe.2021.100164 (2021).

38 Ko, K. K. K. *et al.* Emergence of SARS-CoV-2 Spike Mutations during Prolonged Infection in Immunocompromised Hosts. *Microbiol Spectr* **10**, e0079122, doi:10.1128/spectrum.00791-22 (2022).

39 Marques, A. D. *et al.* SARS-CoV-2 evolution during prolonged infection in immunocompromised patients. *mBio* **15**, e0011024, doi:10.1128/mbio.00110-24 (2024).

40 Choi, B. *et al.* Persistence and Evolution of SARS-CoV-2 in an Immunocompromised Host. *N Engl J Med* **383**, 2291-2293, doi:10.1056/NEJMc2031364 (2020).

41 Kemp, S. A. *et al.* SARS-CoV-2 evolution during treatment of chronic infection. *Nature* **592**, 277-282, doi:10.1038/s41586-021-03291-y (2021).

42 Al-Jasser, F. S., Nouh, R. M. & Youssef, R. M. Epidemiology and predictors of survival of MERS-CoV infections in Riyadh region, 2014-2015. *J Infect Public Health* **12**, 171-177, doi:10.1016/j.jiph.2018.09.008 (2019).

43 Wang, L. F. *et al.* Review of bats and SARS. *Emerg Infect Dis* **12**, 1834-1840, doi:10.3201/eid1212.060401 (2006).

44 National Deer Association, S. An Annual Report on the Status of White-Tailed Deer - The Foundation of the Hunting Industry in North America. (National Deer Association, 2022).

45 Rosenberry, C. R., Fleegle, J. T. & Wallingford, B. D. Monitoring Deer Populations in Pennsylvania. (Bureau of Wildlife Management, Pennsylvania Game Commission, 2011).

46 Hale, V. L. *et al.* SARS-CoV-2 infection in free-ranging white-tailed deer. *Nature* **602**, 481-486, doi:10.1038/s41586-021-04353-x (2022).

47 Marques, A. D. *et al.* Multiple Introductions of SARS-CoV-2 Alpha and Delta Variants into White-Tailed Deer in Pennsylvania. *mBio* **13**, e0210122, doi:10.1128/mbio.02101-22 (2022).

48 Feng, A. *et al.* Transmission of SARS-CoV-2 in free-ranging white-tailed deer in the United States. *Nat Commun* **14**, 4078, doi:10.1038/s41467-023-39782-x (2023).

49 Martins, M. *et al.* From Deer-to-Deer: SARS-CoV-2 is efficiently transmitted and presents broad tissue tropism and replication sites in white-tailed deer. *PLoS Pathog* **18**, e1010197, doi:10.1371/journal.ppat.1010197 (2022).

50 Anis, E. *et al.* Evaluation of a real-time RT-PCR panel for detection of SARS-CoV-2 in bat guano. *J Vet Diagn Invest* **33**, 331-335, doi:10.1177/1040638721990333 (2021).

51 National Agricultural Statistics, S. Pennsylvania Cropland Data Layer 2021. (2021).

52 Smyth, D. S. *et al.* Tracking cryptic SARS-CoV-2 lineages detected in NYC wastewater. *Nat Commun* **13**, 635, doi:10.1038/s41467-022-28246-3 (2022).

53 Caserta, L. C. *et al.* White-tailed deer (Odocoileus virginianus) may serve as a wildlife reservoir for nearly extinct SARS-CoV-2 variants of concern. *Proc Natl Acad Sci U S A* **120**, e2215067120, doi:10.1073/pnas.2215067120 (2023).

54 Magazine, N. *et al.* Mutations and Evolution of the SARS-CoV-2 Spike Protein. *Viruses* **14**, doi:10.3390/v14030640 (2022).

55 Li, Y. *et al.* Antibody landscape against SARS-CoV-2 reveals significant differences between non-structural/accessory and structural proteins. *Cell Rep* **36**, 109391, doi:10.1016/j.celrep.2021.109391 (2021).

56 Emam, M., Oweda, M., Antunes, A. & El-Hadidi, M. Positive selection as a key player for SARS-CoV-2 pathogenicity: Insights into ORF1ab, S and E genes. *Virus Res* **302**, 198472, doi:10.1016/j.virusres.2021.198472 (2021).

57 Nickbakhsh, S. *et al.* Epidemiology of Seasonal Coronaviruses: Establishing the Context for the Emergence of Coronavirus Disease 2019. *J Infect Dis* **222**, 17-25, doi:10.1093/infdis/jiaa185 (2020).

58 Wiemken, T. L. *et al.* Seasonal trends in COVID-19 cases, hospitalizations, and mortality in the United States and Europe. *Sci Rep* **13**, 3886, doi:10.1038/s41598-023-31057-1 (2023).

59 Commission, P. G. White-tailed Deer Wildlife Note. *Pennsylvania Game Commission* (2024).

60 Humphrey, B. The Whitetails' Social Structure. *Mossy Oak Gamekeeper* (2023).

61 Telenti, A. *et al.* After the pandemic: perspectives on the future trajectory of COVID-19. *Nature* **596**, 495-504, doi:10.1038/s41586-021-03792-w (2021).

62 Carpenter, B. *et al.* Stan: A Probabilistic Programming Language. *J Stat Softw* **76**, doi:10.18637/jss.v076.i01 (2017).

63 Adastra, P. A. *et al.* (bioRxiv, 2023).

64 Suchard, M. A. *et al.* Bayesian phylogenetic and phylodynamic data integration using BEAST 1.10. *Virus Evol* **4**, vey016, doi:10.1093/ve/vey016 (2018).

65 Yang, Z. Among-site rate variation and its impact on phylogenetic analyses. *Trends Ecol Evol* **11**, 367-372, doi:10.1016/0169-5347(96)10041-0 (1996).

66 Yang, Z. Maximum-Likelihood Models for Combined Analyses of Multiple Sequence Data. *J Mol Evol* **42**, 587-596, doi:10.1007/BF02352289 (1996).

67 Drummond, A. J., Rambaut, A., Shapiro, B. & Pybus, O. G. Bayesian coalescent inference of past population dynamics from molecular sequences. *Mol Biol Evol* **22**, 1185-1192, doi:10.1093/molbev/msi103 (2005).

68 Ayres, D. L. *et al.* BEAGLE 3: Improved Performance, Scaling, and Usability for a High-Performance Computing Library for Statistical Phylogenetics. *Syst Biol* **68**, 1052-1061, doi:10.1093/sysbio/syz020 (2019).

Figure 1 Click here to access/download;Figure;Fig1.tiff

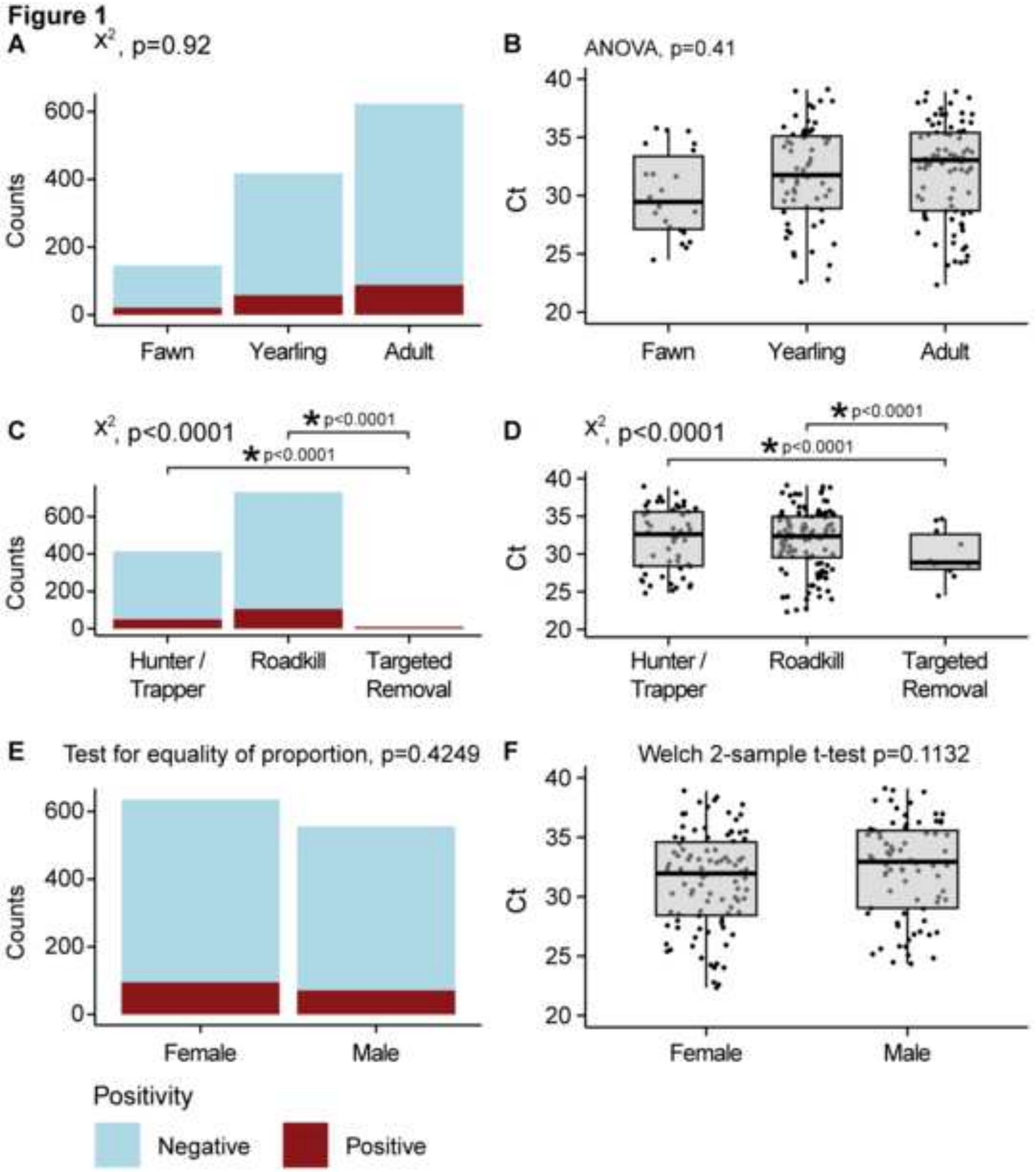

Figure 2

Click here to access/download;Figure;Fig2.tiff

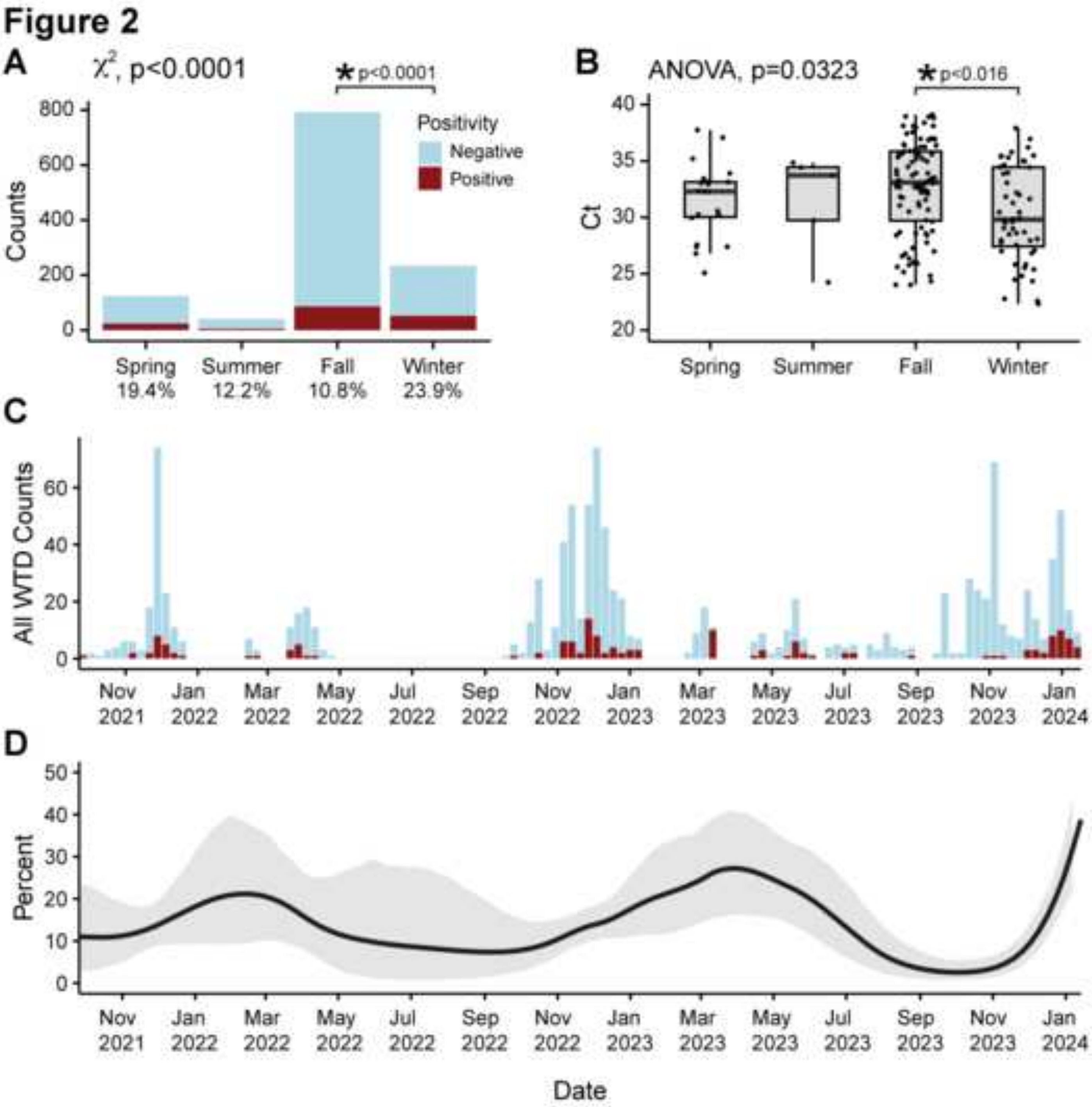

Figure 3

Click here to access/download;Figure;Fig3.tiff

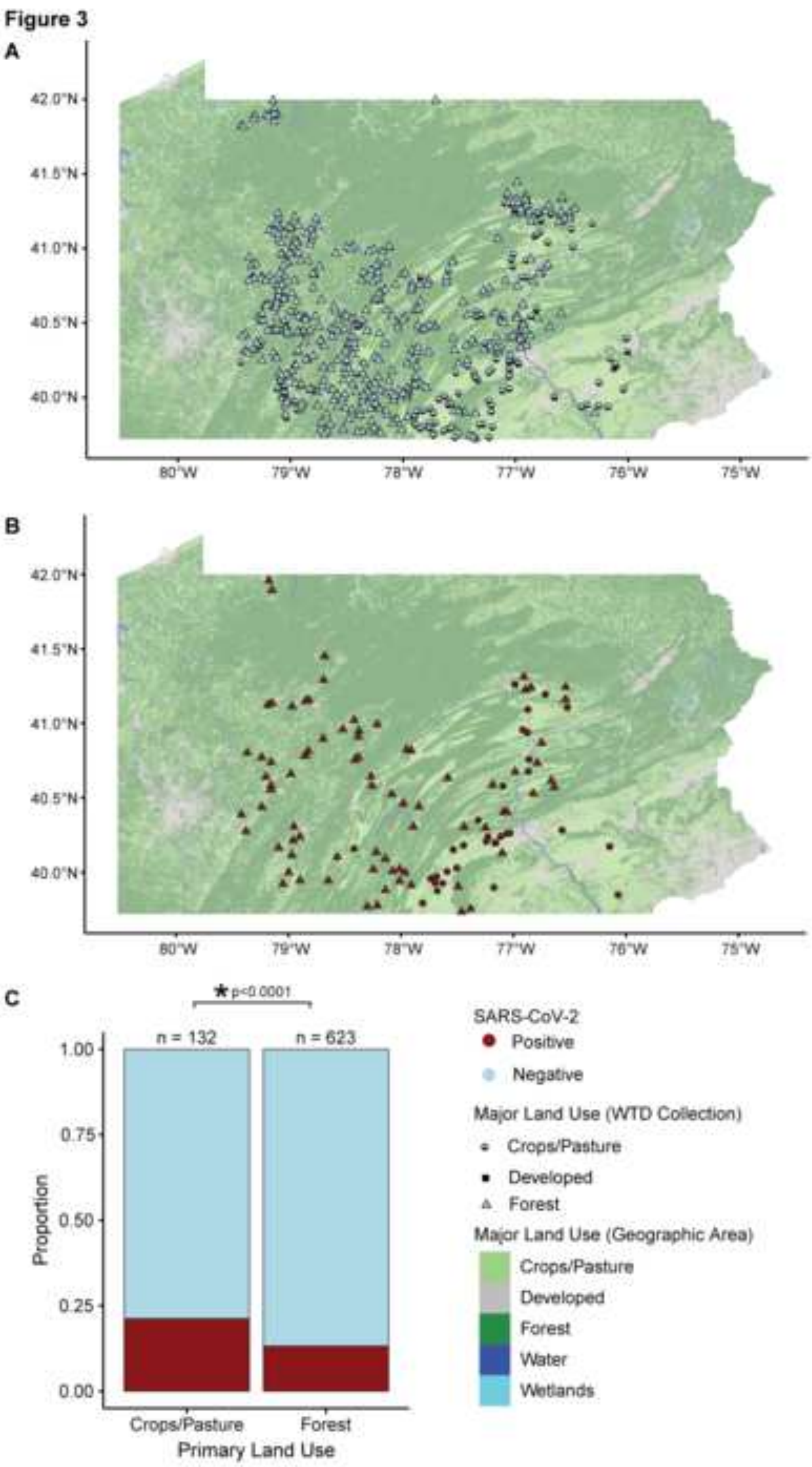

Figure 4

Click here to access/download;Figure;Fig4.tiff

Figure 4

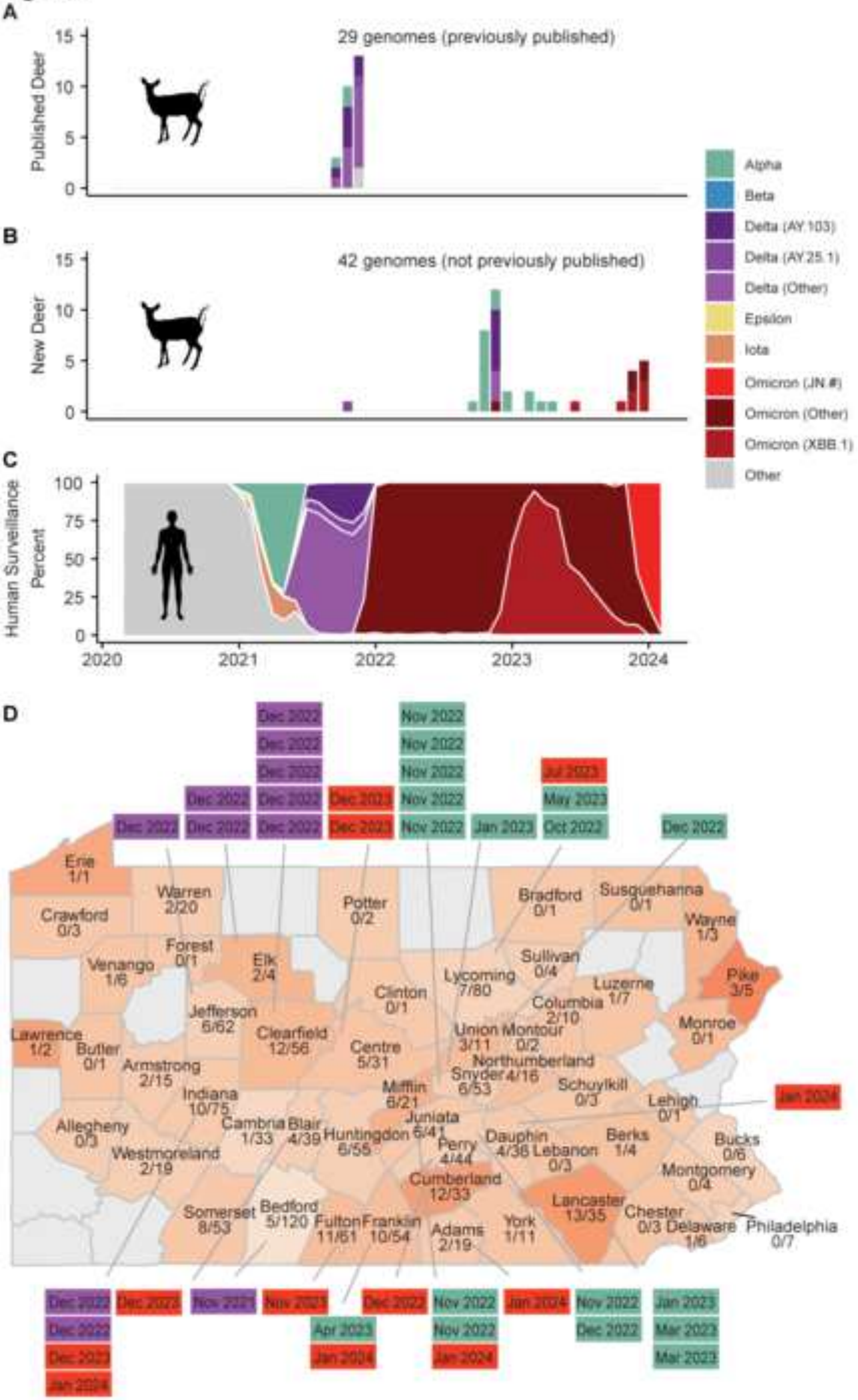

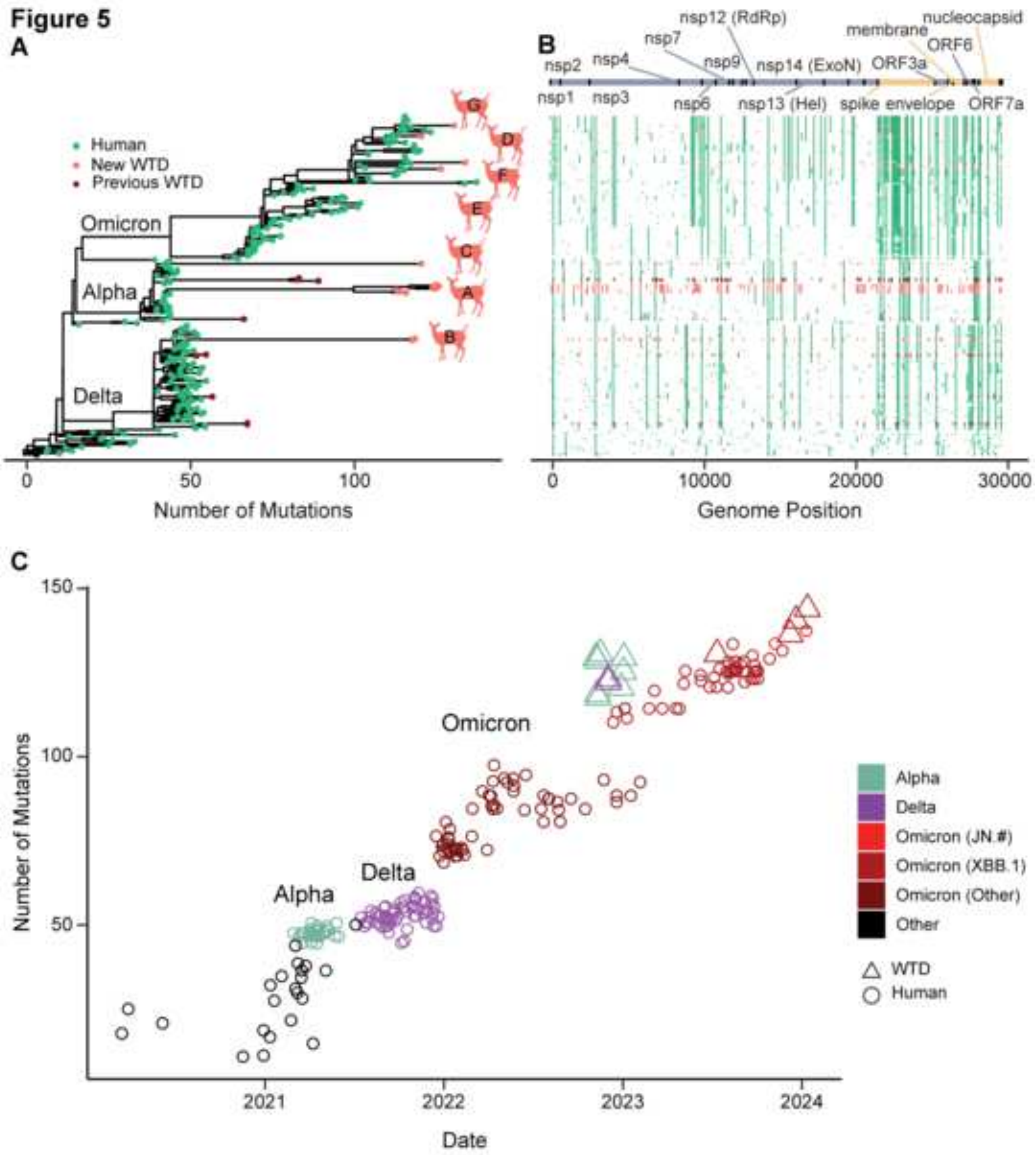
Figure 5
A
Human
New WTD
Previous WTD
Omicron
Alpha
Delta
G
D
F
E
C
A
B
0
50
100
Number of Mutations
B
nsp12 (RdRp)
nucleocapsid
membrane
nsp7
ORF6
nsp2
nsp4
nsp9
nsp14 (ExoN)
ORF3a
nsp1
nsp3
nsp6
nsp13 (Hel)
spike
envelope
ORF7a
0
10000
20000
30000
Genome Position
C
150
100
50
Number of Mutations
Omicron
Delta
Alpha
2021
2022
2023
2024
Date
Alpha
Delta
Omicron (JN.#)
Omicron (XBB.1)
Omicron (Other)
Other
WTD
Human

Figure 6

Click here to access/download;Figure;Fig6.tiff

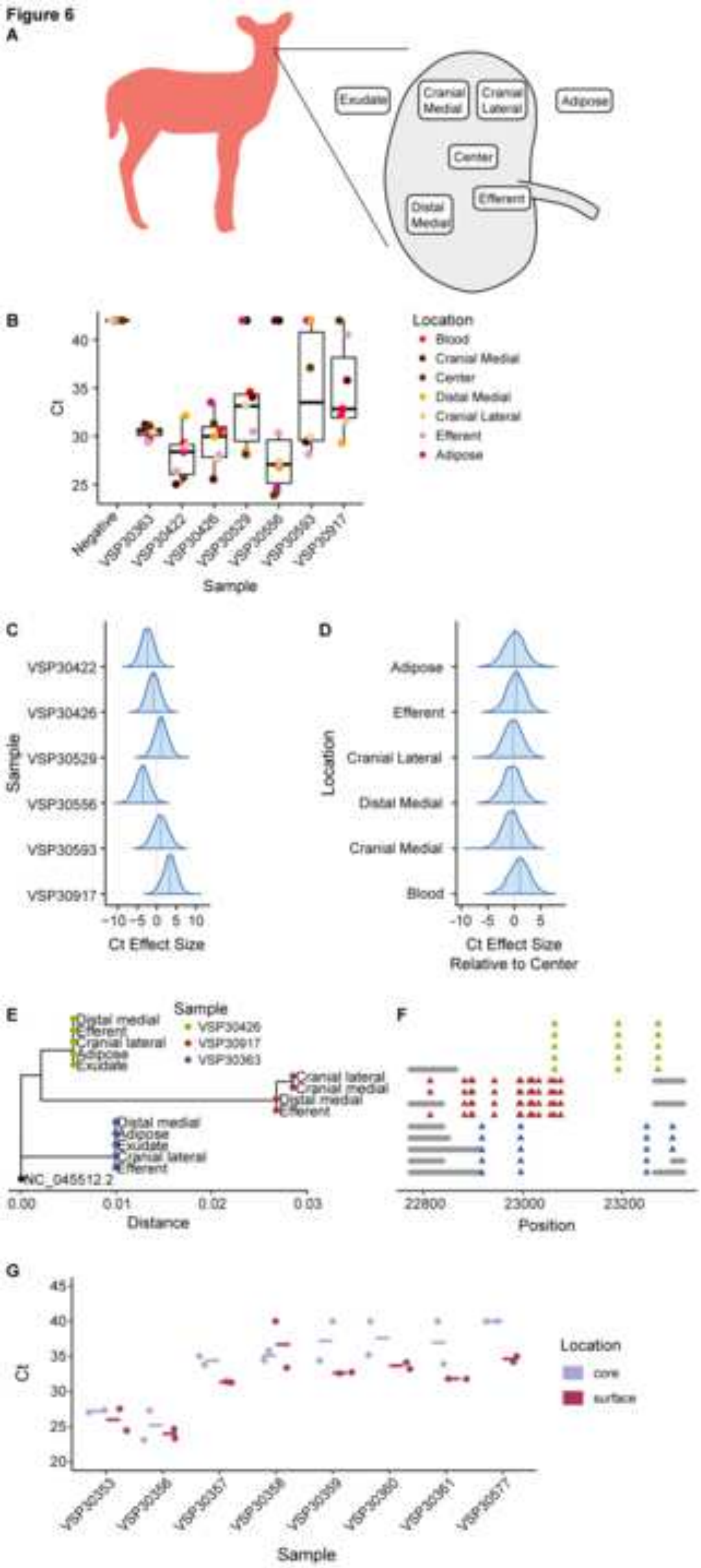

Click here to access/download;Figure;FigS1.tiff

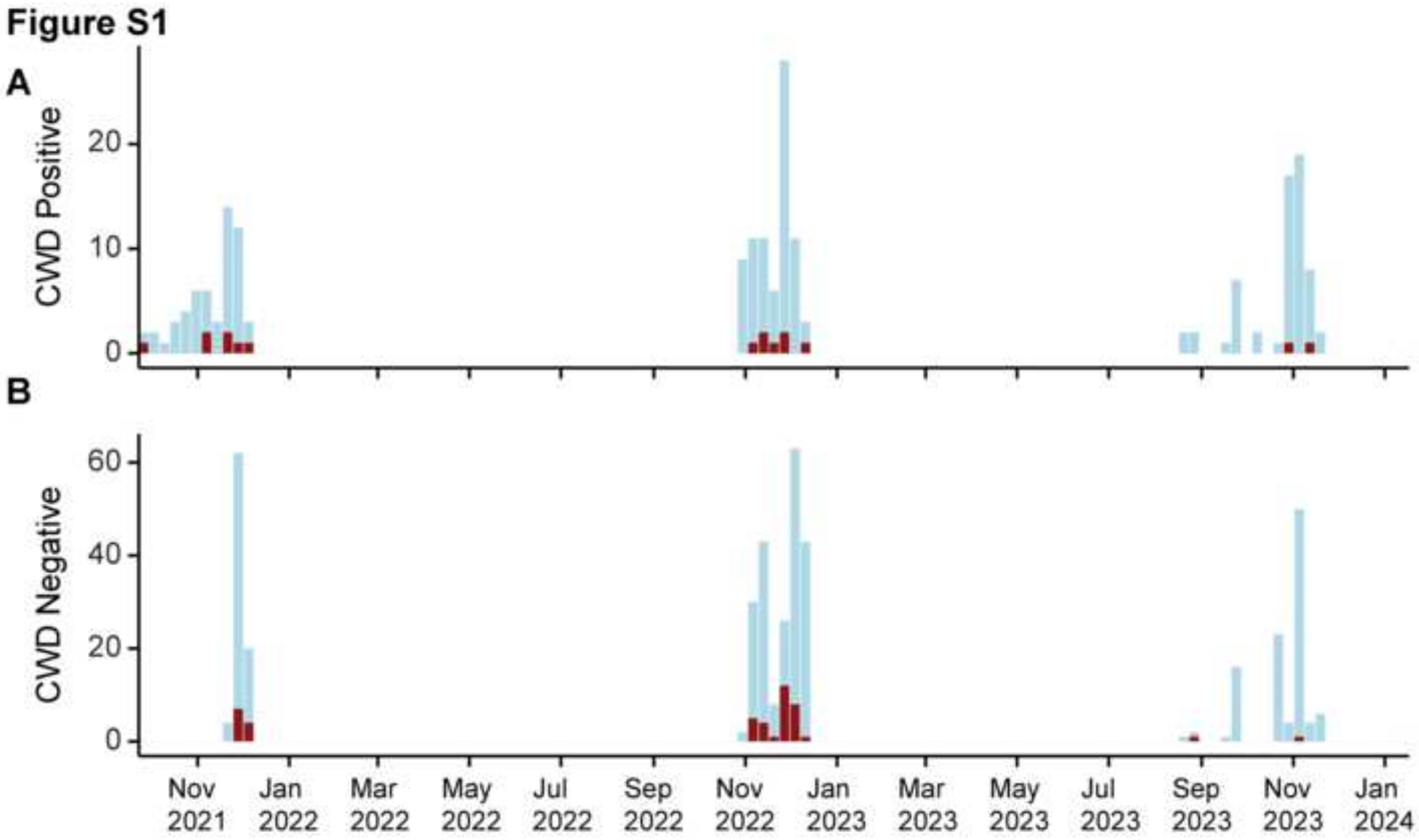

Figure S2 Click here to access/download;Figure;FigS2.tiff

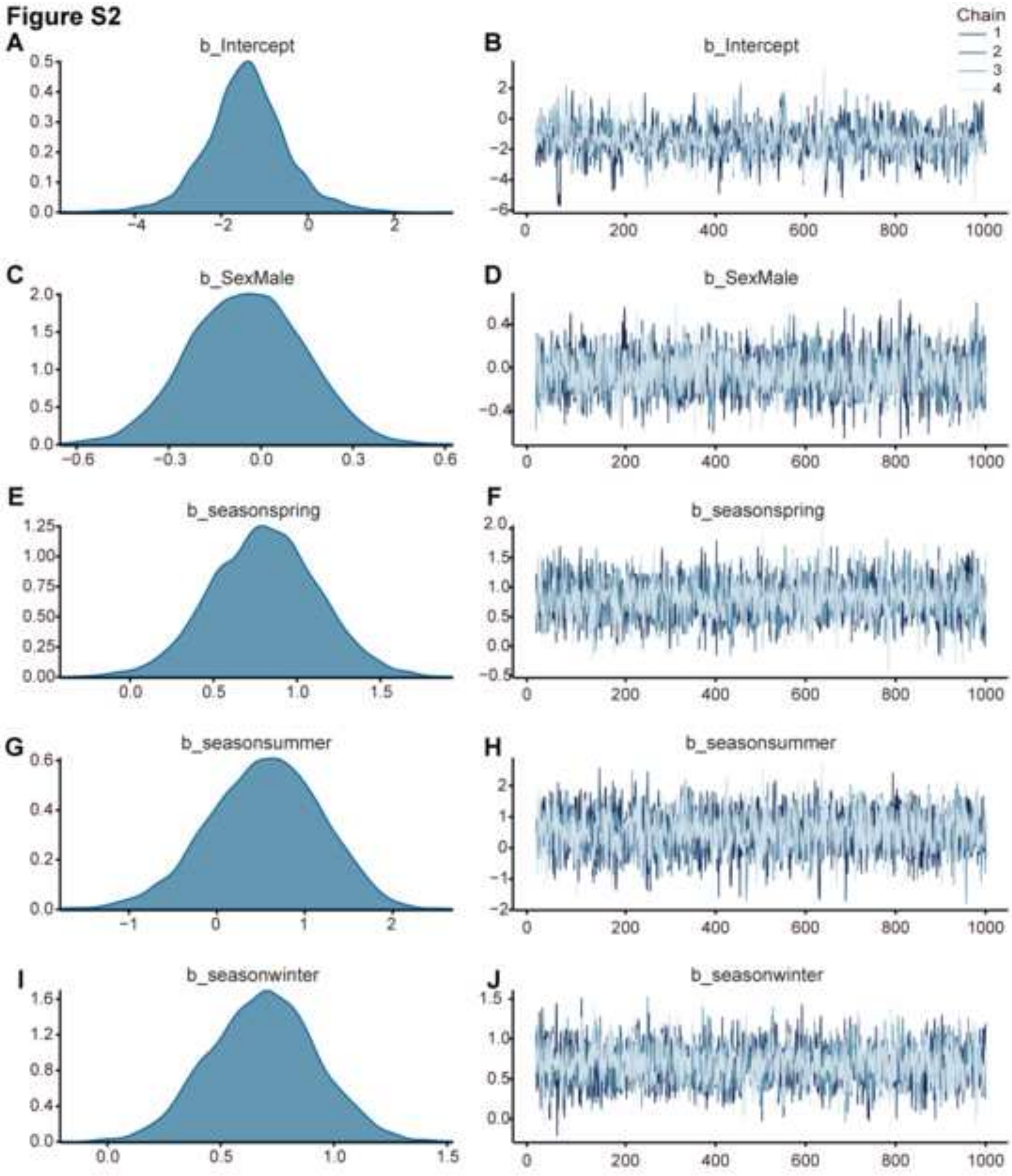

Figure S3

Click here to access/download;Figure;FigS3.tiff

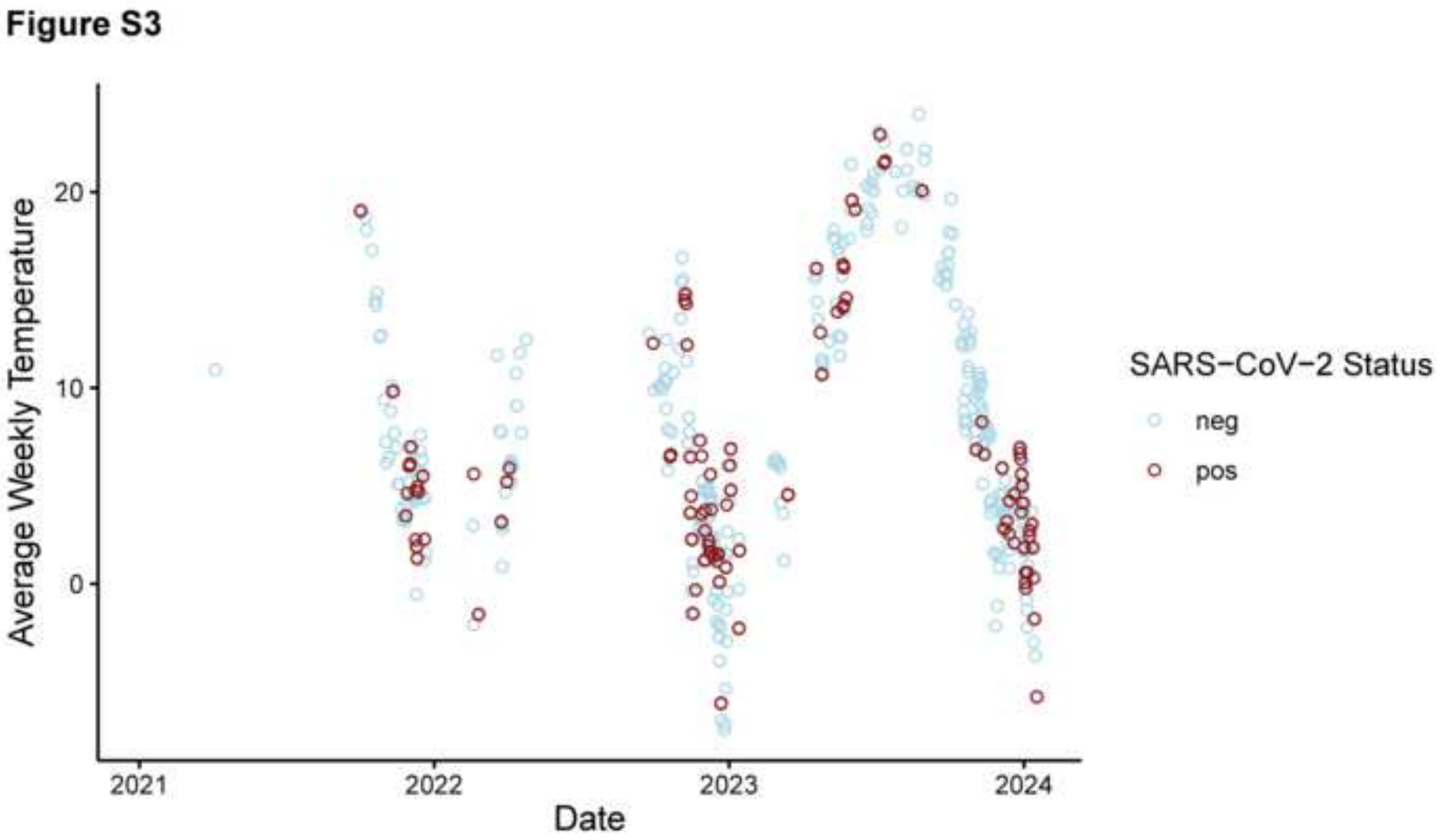

Click here to access/download;Figure;FigS4.tiff

Figure S4

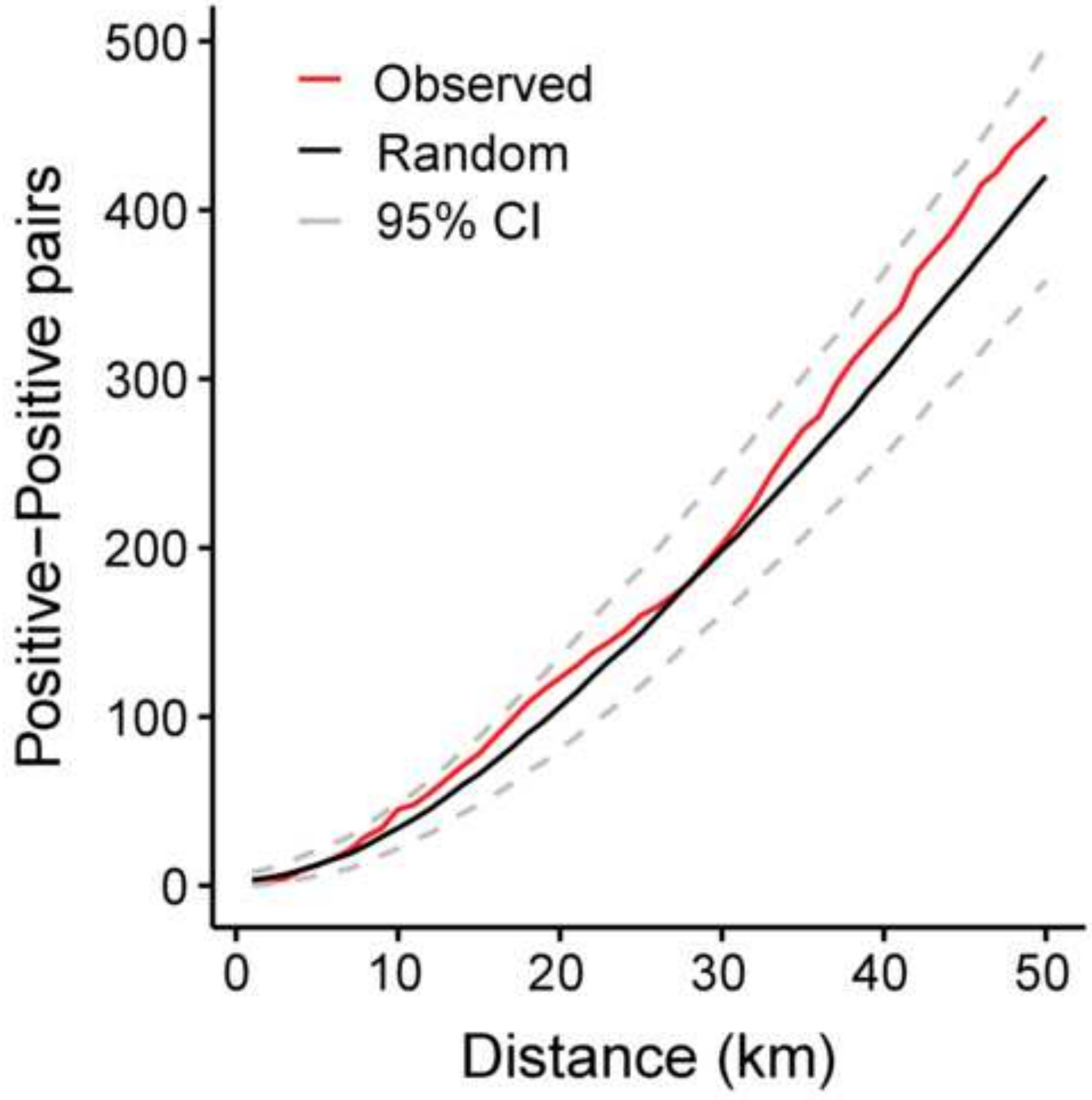

Figure S5

Click here to access/download;Figure;FigS5.tiff

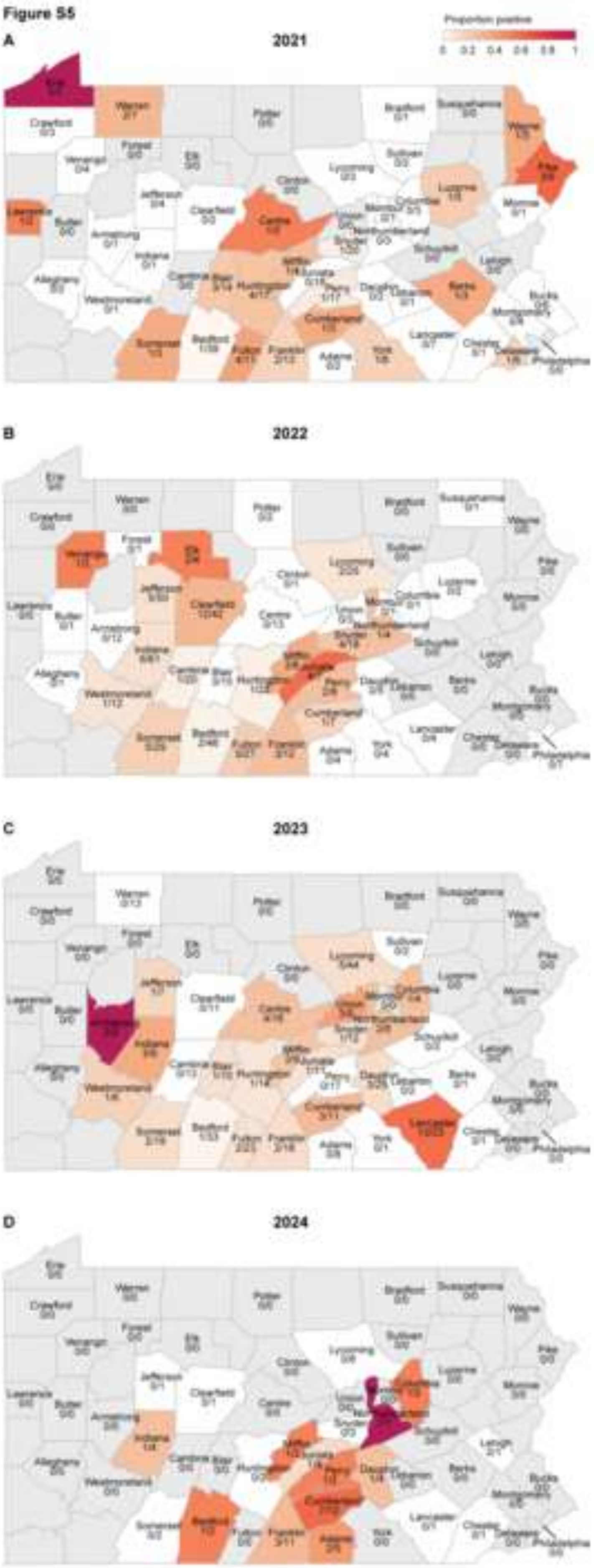

Click here to access/download;Figure;FigS6.tiff

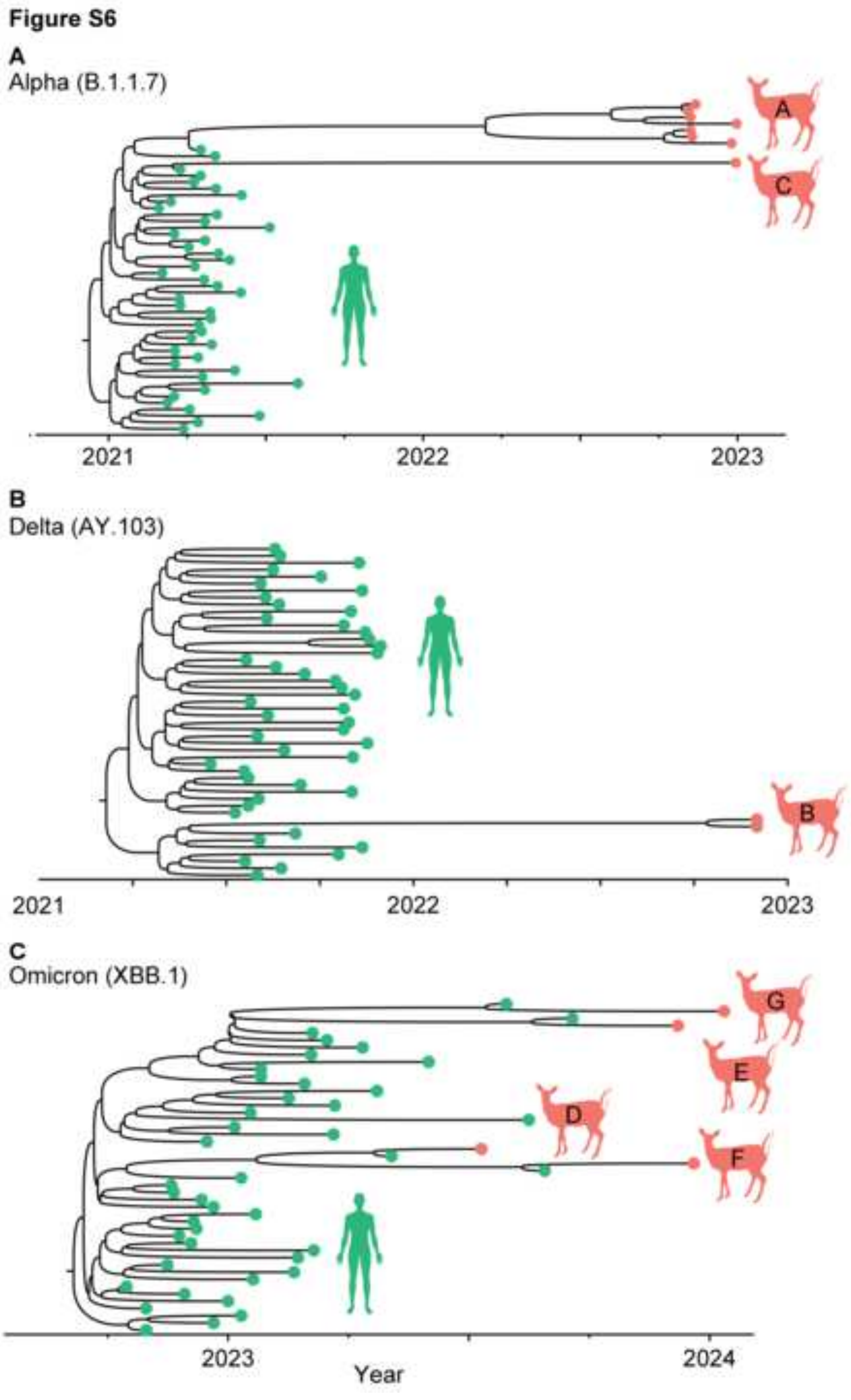

Figure S7 Click here to access/download;Figure;FigS7.tiff

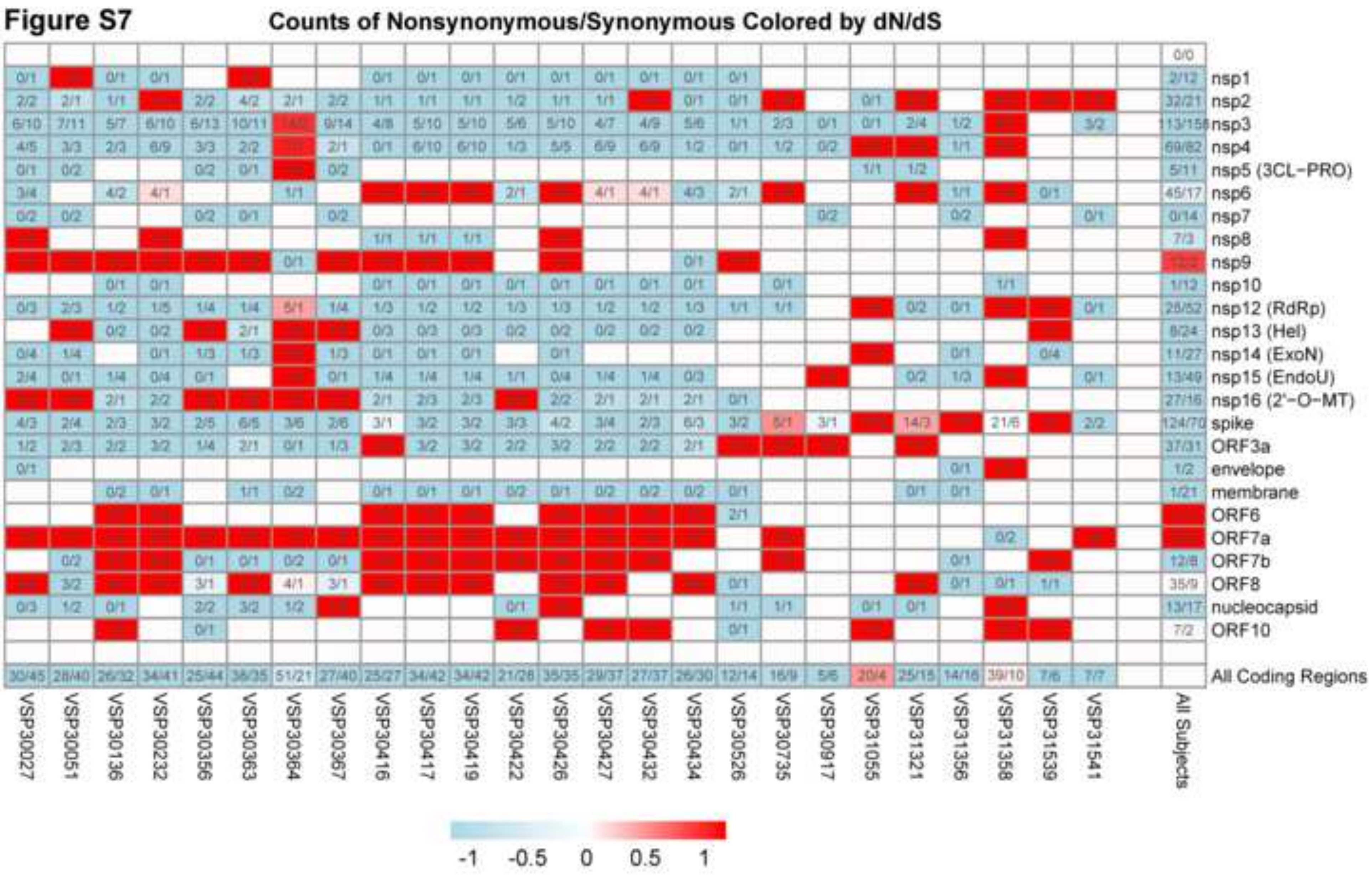

Table S1

Click here to access/download
Supporting Information
TableS1.csv

Table S2

Click here to access/download
Supporting Information
TableS2.csv

Table S3

Click here to access/download
Supporting Information
TableS3.csv

Table S4

Click here to access/download
Supporting Information
TableS4.csv
