# Supplementary material for: Evolution of SARS-CoV-2 in white-tailed deer in Pennsylvania 2021–2024
Source: PLoS Pathog. 2025 Jan 24;21(1):e1012883. doi: 10.1371/journal.ppat.1012883 (PMC11781694; doi:10.1371/journal.ppat.1012883)
Supplement: Attachment — Submitted filename: comments attachment.docx [file ppat.1012883.s010.docx]

**Reviewer comments for manuscript PPATHOGENS-D-24-01513**

**Full Title: Evolution of SARS-CoV-2 in White-tailed Deer in Pennsylvania 2021-2024**

**General comments**

- Infection effect on WTD – there is no information in the introduction or the discussion on the clinical manifestation of SARS-CoV-2 in WTD. If there is any information on this in the literature, it should be referenced.

- The authors make substantial efforts to examine possible correlation or connection between different variables such as age, cause of death, sex, double infection of SARS-COV-2 and	CWD, and geographic location of examined individual WTD. It would be helpful for the reader to understand the rationale for each such examination. What I am missing here is a null hypothesis or a preliminary assumption (prior belief?) regarding the absence or presence of correlation in each case. What do the authors expect to find? What is this expectation based upon? A short explanation regarding each such examination could improve the overall understanding and justify this work.

- It is somewhat confusing to follow the order of results presentation when the figure legends are present before the main text refers to them. I think it would be easier to understand the scientific rationale and to read the manuscript fluently, if the main text will appear continuously, and the figure legends will be placed after it, rather than before the relevant text, as it is now. Many of the figures are complex and contain a lot of data, but their legends are not informative and detailed enough. This makes it difficult to fully understand what was done and what was found or inferred. I made specific comments where I thought is appropriate, and I think that the authors should go through the figure legends and make sure that all axes are labelled when relevant, all signs and symbols are accounted for and all details are explained in full. This way the reader could fully comprehend and appreciate the work.

**Specific comments**

L110 – should be “different sets of primers or different reaction

L115-119 –figure 1: the authors performed elaborated statistical calculations for stratifying the different factors by which the WTD population can be divided into. However, I did not understand whether the sex or cause of death, or the association/dissociation between each of them and Cq values of SARS-COV-2 positive animals, are relevant to the research questions. Please explain how the calculated values presented in panels B, D and F, are relevant (if they are) to the questions of spillback to humans, and the SARS-COV-2 variant dynamics, or explain how are they relevant to the study.

Please explain the relevance and calculation of the P values indicated in the panels.

L163-180 Figure 2 – as in previous data analyses, the authors implement several different statistical calculations. I think that it would be easier for the reader to follow the rationale for each calculation, if the authors would add a sentence or two, describing the considerations for each calculation method, such as chi-square analysis, one- or two-way ANOVA, Tukey’s test, etc.

L192 - Figure S3: I assume that the authors calculate the statistical significance of the difference between incidence rate in temperatures below and above 10C. If they did, it would be more informative to show it. If not, I assume that it would be easy to calculate and show.

L232 - - what is Moran’s I? can the authors add a reference or an explanation?

L266 – can the authors address the fact that only ~25% of the positive samples were “sequencable”, even after nested PCR? Was there any correlation between the initial Cq value and the ability to sequence the virus? Was it something else?

L274 figure 4 – why was the word “in” added to each panel numbering in the legend? In panel D, was it WTD sequences? Human? Both? It is not explicitly stated.

L284-294 – this description includes technical details that I think should be included in the methods section, rather than in the results. The authors should consider revising it to be more concise from the technical aspect.

L311 Figure 5 – it is very difficult for me to understand all the symbols in the heat map, such as the different colors assigned for different genes (structural vs. non-structural?), the black short lines and red short line within the heat map, half way below the genome map, etc. please explain clearly what does each sign and symbol represent.

L346 Figure S6 – add “-“ in “time resolved”. Are the analyses based on complete genome sequencing? Please mention that in the figure legend or in the main text, when referring to the data presented in this figure.

L374 figure S7 – please include a short explanation on the dN/dS analysis, and explain in more details the figure – what does the heat scale (-1 to 1) represent? Are the numbers at the bottom representing individual sequences? Something else? The figure contains a lot of data that need to be more clearly presented to the reader.

L389-407 figure 6 – where is the reference to panels 6E, 6F and 6G? as mentioned in the general comment regarding the null hypothesis that drives each experiment, what did the authors expect to find, when analyzing different tissues from the RPLN? It is likely that the viral load will vary between different parts, even in the same organ, and it is almost certain that it will vary between different animals. So what was the hypothesis here? Additionally, the minor differences in the sequences of VSP30917 tissues (panel 6E, 6F) warrant further explanation, from my experience, it is highly unlikely to find genetic differences of SARS-COV-2 genes within the same organ at a single time point. When e 550bp sequence is examined, this is even more exceptional. I think that the authors should address that either here or in the discussion section. I did not understand what does the short line (rod shape) represent in panel 6G? is it the average of the duplicate? Something else? Please explain.

Discussion section – this study includes a substantial amount of data that is new and important. It raises several important questions that I think should be addressed, at least in short, in the discussion.

First, the possible or empirically effect of SARS-CoV-2 infection on WTD was not mentioned. Are there any data on the clinical effect? Are the animals resistant to the virus? How long does the virus circulate in an individual WTD? Is it enough to accumulate mutations? These questions, in my opinion, are crucial for further understanding of the WTD population as a potential reservoir and source of SARS-CoV-2 spillback.

In this context, are there any hypotheses regarding the transmission from humans to WTD? The virus is not known to survive long periods of time outside the host, and is not known to travel long distances away from an infected person. So the animals need to be in close proximity to infected individuals, in order to be infected. Is there any information in the literature regarding such possible transmission routes?

Another question that comes to mind when reading this study pertains to the serological status of the animals. Is there any information in the literature regarding WTD anti-SARS-CoV-2 antibodies? A survey that will include blood sampling and subsequent serological tests, such as ELISA and neutralization assays, could add important information on exposure of WTD populations to SARS-CoV-2.

I think that the authors should highlight several important points that are derived from the study, and were not emphasized in the discussion. These include the importance of WTD and possibly other animals as both sentinels and reservoir for SARS-CoV-2 and other pathogens, possible routes of spillover transmission that are not yet determined, and the potential of emergence and re-emergence of old and new variants, as a result of non-human circulating. These points could have a major effect on public health policies and should be addressed, at least in short.

Lastly, it was somewhat unusual to sample lymph nodes and blood for SARS-CoV-2. I understand that the availability of the samples dictated this methodology, but I think that since this is not the common practice for detecting SARS-CoV-2 in humans, the authors should also address this, explaining the pros and cons of this sampling methodology, and mentioning the limitations of blood as a sampling material for SARS-CoV-2 viral load assessment. In this respect, if blood samples are available in a condition that allows serum separation and antibody testing, the above comment regarding serology study could be a starting point for a future study.

L532 – can the authors explain why each sample was tested in duplicates? What were the Cq values obtained? Infected animals in acute state should have a low Cq value reflecting high viral load. If the smaples needed to be tested twice for each reaction – that means four different reactions - wouldn't that be excessively unnecessary?

L576 – The entire explanation regarding the Spike-targeted Amplicon Sequencing is lacking details – which regions were amplified? which primer sets were used? What was the first reaction targeting? Within that region, what was targeted in the nested reaction? Were any variant-specific mutations targeted in this procedure? If so, which ones? Please elaborate on the design of the analysis.

L577 – I could not find any primer sequences in table S2. Please provide the table with the primer details.

L612-616 – please add references as links to the reagents supplier relevant website.
