# Supplementary material for: Evolution of SARS-CoV-2 in white-tailed deer in Pennsylvania 2021–2024
Source: PLoS Pathog. 2025 Jan 24;21(1):e1012883. doi: 10.1371/journal.ppat.1012883 (PMC11781694; doi:10.1371/journal.ppat.1012883)
Supplement: Attachment — Submitted filename: MarquesPLOSPathRespv11.docx [file ppat.1012883.s011.docx]

**Department of Microbiology**	Frederic Bushman, Ph.D.

3610 Hamilton Walk	Professor and Chair

426A Johnson Pavilion

Philadelphia, PA 19104-6076

Tel 215.573.8732 Fax 215.573.4856

[bushman@mail.med.upenn.edu](mailto:bushman@mail.med.upenn.edu)

www.med.upenn.edu

October 24, 2024

Re: Response to reviewers’ comments on Manuscript PPATHOGENS-D-24-01513 entitled "Evolution of SARS-CoV-2 in White-tailed Deer in Pennsylvania 2021-2024".

Dear Madam or Sir,

Thanks very much to the reviewers for their helpful comments on our paper PPATHOGENS-D-24-01513 entitled "Evolution of SARS-CoV-2 in White-tailed Deer in Pennsylvania 2021-2024". We have edited the manuscript as suggested, and added additional data. The reviews seem to have come in several pieces, including **Major Issues**, **Minor issues**, **General Comments**, and **Specific Comments**. We list out each below, followed by our responses (indicated in italics).

1) Please provide an Author Summary. This should appear in your manuscript between the Abstract (if applicable) and the Introduction, and should be 150u2013200 words long. The aim should be to make your findings accessible to a wide audience that includes both scientists and non-scientists. Sample summaries can be found on our website under Submission Guidelines:

https://journals.plos.org/plospathogens/s/submission-guidelines#loc-parts-of-a-submission

*An author summary was added after the abstract.*

2) We notice that your supplementary figures are uploaded with the file type 'Figure'. Please amend the file type to 'Supporting Information'. Please ensure that each Supporting Information file has a legend listed in the manuscript after the references list.

*Supplemental figures were changed from “Figures” to be “Supporting information.”*

3) We notice that your supplementary Figures are included in the manuscript file. Please remove them and upload them with the file type 'Supporting Information'. Please ensure that each Supporting Information file has a legend listed in the manuscript after the references list.

*Figures were checked to not be found in the manuscript. Figure legends were moved to be found immediately after their first reference in the manuscript.*

4) Some material included in your submission may be copyrighted. According to PLOSu2019s copyright policy, authors who use figures or other material (e.g., graphics, clipart, maps) from another author or copyright holder must demonstrate or obtain permission to publish this material under the Creative Commons Attribution 4.0 International (CC BY 4.0) License used by PLOS journals. Please closely review the details of PLOSu2019s copyright requirements here: PLOS Licenses and Copyright. If you need to request permissions from a copyright holder, you may use PLOS's Copyright Content Permission form.

Please respond directly to this email and provide any known details concerning your material's license terms and permissions required for reuse, even if you have not yet obtained copyright permissions or are unsure of your material's copyright compatibility. Once you have responded and addressed all other outstanding technical requirements, you may resubmit your manuscript within Editorial Manager.

Potential Copyright Issues:

i) Figures 4, 5, Figure S7, and Figure S8. Please confirm whether you drew the images / clip-art within the figure panels by hand. If you did not draw the images, please provide (a) a link to the source of the images or icons and their license / terms of use; or (b) written permission from the copyright holder to publish the images or icons under our CC BY 4.0 license. Alternatively, you may replace the images with open source alternatives. See these open source resources you may use to replace images / clip-art:

- https://commons.wikimedia.org

- https://openclipart.org/.

ii) Figures 3, 4, and Figure S5. Please (a) provide a direct link to the base layer of the map (i.e., the country or region border shape) and ensure this is also included in the figure legend; and (b) provide a link to the terms of use / license information for the base layer image or shapefile. We cannot publish proprietary or copyrighted maps (e.g. Google Maps, Mapquest) and the terms of use for your map base layer must be compatible with our CC BY 4.0 license.

Note: if you created the map in a software program like R or ArcGIS, please locate and indicate the source of the basemap shapefile onto which data has been plotted.

If your map was obtained from a copyrighted source please amend the figure so that the base map used is from an openly available source. Alternatively, please provide explicit written permission from the copyright holder granting you the right to publish the material under our CC BY 4.0 license.

If you are unsure whether you can use a map or not, please do reach out and we will be able to help you. The following websites are good examples of where you can source open access or public domain maps:

* U.S. Geological Survey (USGS) - All maps are in the public domain. (http://www.usgs.gov)

* PlaniGlobe - All maps are published under a Creative Commons license so please cite u201cPlaniGlobe, http://www.planiglobe.com, CC BY 2.0u201d in the image credit after the caption. (http://www.planiglobe.com/?lang=enl)

* Natural Earth - All maps are public domain. (<http://www.naturalearthdata.com/about/terms-of-use/>).

*Figures 3, 4, 5, S5, and S7 were confirmed to use images, clipart, or map traces that are permitted to be used in submission. Details regarding image sources are found below.*

- *(1) copyright policy: all images are licensed for our use in publication.*
  - *Figure 3 (PA Map):*
    - *Direct link to base map: https://www.pasda.psu.edu/uci/DataSummary.aspx?dataset=2514*
    - *Direct link to license for base map: https://www.usda.gov/policies-and-links#:~:text=Digital%20Rights%20and%20Copyright,%22U.S.%20Department%20of%20Agriculture.%22*
  - *Figure 4 (Deer Image):*
    - *Direct link to image: https://www.phylopic.org/images/6038e80c-398d-47b2-9a69-2b9edf436f64/odocoileus-virginianus*
    - *Direct link to license: https://creativecommons.org/licenses/by-nc/3.0/*
  - *Figure 4 (Human Image):*
    - *Direct link to image: https://www.phylopic.org/images/036b96de-4bca-408e-adb6-2154fcd724ef/homo-sapiens-sapiens*
    - *Direct link to license: https://creativecommons.org/licenses/by/4.0/*
  - *Figure 5 (PA Map):*
    - *Direct link to image: https://cran.r-project.org/web/packages/maps/index.html and https://github.com/adeckmyn/maps/tree/master/data*
    - *Direct link to license: https://cran.r-project.org/web/licenses/GPL-2*
  - *Figure S5 (PA Map):*
    - *Direct link to image: https://cran.r-project.org/web/packages/maps/index.html and https://github.com/adeckmyn/maps/tree/master/data*
    - *Direct link to license: https://cran.r-project.org/web/licenses/GPL-2*
  - *Figure S7 (Deer Image):*
    - *Direct link to image: https://www.phylopic.org/images/9df2ed00-b918-4d3a-82bd-1a94283f7c0f/odocoileus-virginianus*
    - *Direct link to license: https://creativecommons.org/licenses/by/4.0/*
  - *Figure S8 (Deer Image):*
    - *Direct link to image: https://www.phylopic.org/images/6038e80c-398d-47b2-9a69-2b9edf436f64/odocoileus-virginianus*
    - *Direct link to license: https://creativecommons.org/licenses/by-nc/3.0/*
  - *Figure S8 (Human Image):*
    - *Direct link to image: https://www.phylopic.org/images/036b96de-4bca-408e-adb6-2154fcd724ef/homo-sapiens-sapiens*
    - *Direct link to license: https://creativecommons.org/licenses/by/4.0/*

5) We note that your Data Availability Statement is currently as follows: "All relevant data are within the manuscript and its Supporting Information files.". Please confirm at this time whether or not your submission contains all raw data required to replicate the results of your study. Authors must share the “minimal data set” for their submission. PLOS defines the minimal data set to consist of the data required to replicate all study findings reported in the article, as well as related metadata and methods (https://journals.plos.org/plosone/s/data-availability#loc-minimal-data-set-definition).

For example, authors should submit the following data:

1) The values behind the means, standard deviations and other measures reported;

2) The values used to build graphs;

3) The points extracted from images for analysis..

Authors do not need to submit their entire data set if only a portion of the data was used in the reported study.

If your submission does not contain these data, please either upload them as Supporting Information files or deposit them to a stable, public repository and provide us with the relevant URLs, DOIs, or accession numbers. For a list of recommended repositories, please see https://journals.plos.org/plosone/s/recommended-repositories.

If there are ethical or legal restrictions on sharing a de-identified data set, please explain them in detail (e.g., data contain potentially sensitive information, data are owned by a third-party organization, etc.) and who has imposed them (e.g., an ethics committee). Please also provide contact information for a data access committee, ethics committee, or other institutional body to which data requests may be sent. If data are owned by a third party, please indicate how others may request data access.

*We confirm that all the data used in this manuscript is available in the supplemental files or the manuscript. See data availability section for more information.*

6) Please ensure that the funders and grant numbers match between the Financial Disclosure field and the Funding Information tab in your submission form. Note that the funders must be provided in the same order in both places as well.

Currently, order of grants are different in both places.

*We checked to ensure that the list of funding sources matches in the Financial Disclosure and Funding Information tab.*

7) Please amend your detailed Financial Disclosure statement. This is published with the article, therefore should be completed in full sentences and contain the exact wording you wish to be published.

*We checked to ensure statements are written in the exact wording we wish to publish.*

8) Thank you for stating "None of these sponsors/funders played a role in the study design, data collection, analysis, decision to publish, or preparation of the manuscript."

Please amend your detailed Financial Disclosure statement. This is published with the article, therefore should be completed in full sentences and contain the exact wording you wish to be published.

i). If the funders had no role in your study, please state: “The funders had no role in study design, data collection and analysis, decision to publish, or preparation of the manuscript.”

ii). If any authors received a salary from any of your funders, please state which authors and which funders.

*We revised “None of these sponsors/funders played a role in the study design, data collection, analysis, decision to publish, or preparation of the manuscript” to state “The funders had no role in study design, data collection and analysis, decision to publish, or preparation of the manuscript.” No authors received salary from the funders.*

**Major Issues**

Reviewer 1. None

Reviewer 2. None

Reviewer 3. The molecular analyses – real-time PCR, Sanger sequencing and Illumina genomic sequencing, are key techniques that were crucial for obtaining the data. Subsequently, the statistical and bioinformatic methodologies that were used, such as applying Bayesian regression model to test connection between different study parameters, using Time-Resolved Phylogenetic Analysis for genomic sequences, were essential for the data analysis in this study. The combined use of these approaches enabled interpretation and inference of the collected data.

*These were indeed the methods used.*

Reviewer 4. A substantial proportion of samples present Ct values ≤35. To which extend can we talk about positivity resulting from a productive infection vs any potential environmental or experimental contamination?

*In the revised manuscript we present a more detailed description of results with our negative controls. We believe our methods were extremely rigorous: (1) negative controls were required to not have amplification for 45 cycles or more, (2) two negative controls were included for each batch including a negative control that underwent RNA extraction, (3) for a sample to be considered positive we required three of four RT-qPCR tests to show amplification for our samples (two replicates of two independent sequence targets in the SARS-CoV-2 genome). Protocols were carried out in different rooms and hoods designated for each step of the procedure (i.e. RNA extraction and RT-qPCR prep occurred in two different hoods and library preps were performed in different rooms). Downstream reagents were never stored with upstream reagents. Altogether, this gives us confidence that our standards of rigor and protocols minimized environmental and experimental contamination.*

- Figure 5: I might be wrong, but this figure does not seem to be called in the text when its result are presented.

*We have now called out to this figure in the revised draft.*

It would be really interesting to present aa mutational profiles alongside genomic location of SNPs. The reader might find himself left with a bitter taste in the mouth after expecting to find a mine of information which could rapidly be translated into further investigations in the community.

*We have added a new figure panel to the manuscript. In new figure 6 we map nucleotide mutations and compare them to AA (nonsynonymous) mutations in the SARS-CoV-2 genome. We expand on the dN/dS analysis to fill this gap.*

A focus on spike mutational profiles looks obvious given its important in driving immune evasion, tissue tropism and virulence and since the authors targeted the RBD region but also interesting to present for others such as nsp6, ORF6, and N. Comparative approaches with what’s described in the human population would be beneficial, if possible. Comparing with Omicron BA.1 and BA.2.86 (both very likely resulting from saltation events over the course of chronic infection or local circulation within a given population) could be meaningful to potentially identify patterns or hotspots of mutations regardless of the species.

*We have added figure 6 to address mutational hot spots throughout the genome including RBD region and nsp6, ORF6, N, and others. We also added an additional paragraph in the results section from which WTD are compared to their nearest genetic human-derived isolates to look for regions that are hotspots after spilling over to WTD.*

- lines 318-324, Do the results really support the conclusion suggested by the authors? Are the conditions well controlled to discriminate between recent versus ancient/long term infections? Do we have the information of the percentage of animals having such highly mutated sequences? In other words, could we affirm that the most highly mutated sequences do spread/circulate in WTD population or is it coming from a very small percentage of samples individuals? Could we expect chronic/long infection in WTD?

*The text has been revised to avoid confusion. We do not intend to suggest we have unambiguously proven chronic/long term infection of WTD. The phrase “long-term onward spread of SARS-CoV-2 in WTD” was intended to communicate that the WTD population maintained SARS-CoV-2 virus (unsampled) for a long duration of time, not that individual WTD necessarily maintained persistent infection.*

- Line 342-343 and likely Figure 5: the authors conclude that “the virus did not diverge at significantly faster rate than observed in human-to-human transmission”. This is different from McBride et al. Nature Communications 2023 where they estimated the evolution rate to be 3 times faster in WTD compared to human. And I’m surprised that previous study is not cited and discussed in the manuscript. What could explain this difference in conclusions reached?

*In the revised manuscript we cite the McBride paper and discuss the measured mutation rates. The discrepancy between the reported evolution rates could be due to (1) the time-frame of sampling and/or (2) method of estimating rates. McBride sampled the deer with spillover estimating 5-8 months before collection. (1) In our paper, we filtered our analysis to have only the highest quality (98% sequence coverage)_that phylogenetically presented as chains of transmission which resulted in estimated spillover that of more than 18 months. Consequently, evolution rates could be nonlinear with higher rates in WTD in the first months after species introduction and slow later, similar to that of humans. Moreover, McBride uses evolution rate calculations that infer the substitution rate over the 5-8 months between the last human and first deer sequence. This assumes that there are no intermediate hosts between the human-to-deer transmission. In our analysis, we restrict the timeline to the reported chain of transmission (from the first sampled deer to the most recent sampled deer). This results in a smaller window of prediction but it no longer requires the assumption that there were no intermediate hosts. We believe that this number most accurately reflects the evolution rate in deer once deer become a stable reservoir.*

*Both McBride and our estimates are not in conflict since we measure rates once established in deer populations for more than 18 months while McBride measured rates including at species spillover, which also could captured effects from intermediate host species.*

**Minor Issues**

Reviewer 1. Line 56: I think it would be more informative if the authors can add the geographical information to the Supp Tables 1. For ex, a column of the countries where the animals were captured.

*The suggested data has been added to the supplementary table.*

Line 94: How do the authors think a prion disease could potentially trigger a new SARS-CoV-2 variant? Since the readers are likely naïve to the prion disease in WTD, it would be helpful to explain how it effects the general health of deer and if it weakens the immune system so that they are more prone to other infections such as SAR-Cov-2. Also, the authors should mention here if deer show any SARS-CoV-2 symptoms in their study and in previous reports?

*As suggested, we have expanded the background on the prion disease, and also presented more background on the pathogenesis of SARS-CoV-2 in deer.*

Line 104: Please provide the meanings of abbreviations used in the Supp Table 2 where applicable to make understanding clear for wider readers. It would also be useful to explain terms such as “n1_half_positive”, “hict_lineage” etc. These can be added somewhere in the Table or header.

*In the revised draft we have added definitions for the abbreviations in Supp Table 2 and the indicated terms. This is added to the manuscript in supplemental material “Supp Table 2” description.*

Line 151: Please mention the red represents the SARS-CoV-2 positive samples and the blue represents the negative ones.

*We have added explanations of the colors in the labels as suggested.*

Line 275: Please cite the previous publications.

*We have added the indicated citations to the revised text.*

Figure 5: This figure is not cited in the Text! What is the reference to calculate the Mutation numbers? Can you please provide the acc. number of the reference in the text? I wanted to note that the provided resolution of the Figure 5 didn’t let me to see the colors and lineages clearly, especially for Fig5A.

*We now call out to Figure 5 from the text. We have improved the resolution of the figure, and added the suggested reference. If the resolution is bad in the draft, then this may be compression done by the journal. Please use their “Click here to access/download;Figure;Fig5.tiff” link at the top of the page to download the full submitted 4016x4659 image.*

Line 283: Did the authors identify recurring amino acid substitutions among the WTD isolates?

*Yes—we did find a recurrent amino acid substitution, and this is now mentioned in the text.*

Line 320: The lack of previous isolates in the recent samples contradict the idea of the WTD being a reservoir. Do you think that it is due to the number of samples?

*We do believe that the failure to recover older lineages in new samples is probably due to sparse sampling, but of course this is not the only possibility. We now discuss this in the revised manuscript.*

Line 305: Please indicate the acc. Numbers of the isolates used to create the phylogenetic in Figure 5A. I think Table S2 must be cited. Please clarify in Table S3 which sequences are from WTD.

*We have made the attributions clearer in the revised manuscript and updated Table S3 (Now table S4) to indicate which isolates were derived from WTD.*

Lines 309-310: Please site the previous works.

*We have added the indicated citations.*

Line 442: Please cite the “previous data”.

*We have added the indicated citations.*

Line 445: I think spillover from humans to WTD begs more discussion. What could be the route of transmission between WTD and humans? This can be about human behavior. How often humans and WTD occupy the same environment? What kind of activities humans do in WTD occupied regions in the sampled places? Do humans get in close contact with WTD or for how long SAR-CoV-2 can remain in the air according to the published studies?

*We have added a speculative paragraph on possible spillover mechanisms to the revised draft.*

Line 451: If WTD became a reservoir for Alpha and Delta variant, why more recent isolates don’t have these variants?

*As mentioned above, we have added a discussion of possibilities to the revised manuscript.*

Line 608: “primers specific to the study” please be more specific and cite.

*We referenced the supplemental table containing reference numbers for the ARTIC Primers sold by IDT used in the whole genome sequencing section.*

Reviewer 2.

Throughout paper: Suggest switching to standard date format “yyyy/mm/dd”.

*We have considered this suggestion and have opted for the North American standard using mm/dd/yyyy because the sampling and immediate impacts from the paper were observed in the United States and apply to the United States and Canada.*

Line 300: replace “out” with “our”.

*We have replaced the typo.*

Line 519 and 536: replace “doped in/doping in” with “spiked in/spiking in”.

*We have made the suggested edits.*

Line 595: Please clarify what is meant by “no notable SARS-CoV-2 sequences were detected” when results for the sequencing are presented on p13.

*The text was clarified to the intended meaning.*

Ref 63 has been published already – please amend (doi: 10.1371/journal.pone.0294283)

*We now call out to the published paper.*

Line 650: “MCMC” defined in this line, but the abbreviation is not always used in the text (where the full name is used; e.g. line 544).

*We now define the abbreviation and use it consistently.*

Reviewer 3.

The order in which the figure legends are placed within the main text is confusing and makes it difficult to follow the flow of the manuscript. I suggest that the main text will be organized as continuous flow of paragraphs, and the figure legends will be placed subsequently. Of course, in the finalized version, the legends will be placed next to the figures, so this should be easier to follow.

*Thank you for the suggestion. We have been following the journal’s guidelines.*

My other comment regarding the legends, is the need to provide more detailed explanations regarding the different panels in each figures, to improve clarity. As many of the figures are complex and contain several panels, a more detailed explanation could be helpful.

*As suggested, we have added more detail to the figure legends.*

Lastly, I think that placing the methods section before the results makes more sense, but the Editors and Journal policy dictate this.

*Again, we have been following the journal’s guidelines.*

Reviewer 4.

- The sample distribution in Fig.S3 seems to be showing predictive effect of temperature on positivity with apparently a higher abundance of positive samples at temperatures ≤ 10°C despite the text stating it did not. Could the authors double check the analysis?

*Thank you for the comment, we checked this and added a new supplemental figure S3B and found no significant difference p=0.25.*

- lines 215-222, how does this correlate with prevalence in human according to the different seasons?

*We include in the discussion a paragraph regarding seasonality in humans compared to white-tailed deer. The basis of seasonality in humans is uncertain and intertwined with the origin of new variants. Thus there is no simple relationship with seasonality so we unable to compare seasonality in humans to seasonality in deer.*

- Figure 5: Use different colours between new WTD sequences and those from previous work. It is difficult to distinguish between both groups otherwise.

*We have modified the colors as suggested to indicated the different groups.*

- Line 328, Could this be due to XBB.1 lineage having gained/increased capacity to infect deer?

*We believe this to be possible and likely. Text has been added to the discussion to present this as a potential hypothesis for future work.*

- Line 389-436 and Figure 6: I am not entirely sure what to think about this section and associated results. It sounds more like nice supplementary data and findings which were performed as control rather than being critical for the study and conclusions. Especially if placed in front of the mutational signatures observed in WTD compared to human which lack in this manuscript, as mentioned before.

*We have moved old figure 6 to the supplement as suggested (now figure S7).*

- Line 445, “how this is happening so efficiently…” maybe ton down that statement as it is not clear yet how efficient this is versus rare spillovers followed by efficient spread once in WTD populations.

*We have reworded the indicated section.*

- Line 455-470, some statements and hypotheses are popping without results directly linked to them and analysis of correlation are missing.

*These hypotheses are more speculative and includes ideas that we do not have data to test. We think these speculations are useful to include because they help formulate hypothesis for future studies.*

**General comments**

- Infection effect on WTD – there is no information in the introduction or the discussion on the clinical manifestation of SARS-CoV-2 in WTD. If there is any information on this in the literature, it should be referenced.

*We have added text to the introduction to address the clinical manifestation of SARS-CoV-2 in WTD.*

- The authors make substantial efforts to examine possible correlation or connection between different variables such as age, cause of death, sex, double infection of SARS-COV-2 and CWD, and geographic location of examined individual WTD. It would be helpful for the reader to understand the rationale for each such examination. What I am missing here is a null hypothesis or a preliminary assumption (prior belief?) regarding the absence or presence of correlation in each case. What do the authors expect to find? What is this expectation based upon? A short explanation regarding each such examination could improve the overall understanding and justify this work.

*We have added text to describe more fully the hypothesis that were tested.*

- It is somewhat confusing to follow the order of results presentation when the figure legends are present before the main text refers to them. I think it would be easier to understand the scientific rationale and to read the manuscript fluently, if the main text will appear continuously, and the figure legends will be placed after it, rather than before the relevant text, as it is now. Many of the figures are complex and contain a lot of data, but their legends are not informative and detailed enough. This makes it difficult to fully understand what was done and what was found or inferred. I made specific comments where I thought is appropriate, and I think that the authors should go through the figure legends and make sure that all axes are labelled when relevant, all signs and symbols are accounted for and all details are explained in full. This way the reader could fully comprehend and appreciate the work.

*The order of the figures have been adjusted to reference the material in the text before the figures are presented.*

*More information describing the figures is now provided in the legends.*

*Axes were checked and in some cases modified to ensure that they had adequate labels.*

**Specific comments**

L110 – should be “different sets of primers or different reaction

*We have edited the indicated suggestion.*

L115-119 –figure 1: the authors performed elaborated statistical calculations for stratifying the different factors by which the WTD population can be divided into. However, I did not understand whether the sex or cause of death, or the association/dissociation between each of them and Cq values of SARS-COV-2 positive animals, are relevant to the research questions. Please explain how the calculated values presented in panels B, D and F, are relevant (if they are) to the questions of spillback to humans, and the SARS-COV-2 variant dynamics, or explain how are they relevant to the study.

Please explain the relevance and calculation of the P values indicated in the panels.

*Figures A, C, and E present the data as binary outcomes while B, D, and F present the analysis of the values as continuous variables. It seems that either view of the data would be incomplete so we have included both.*

L163-180 Figure 2 – as in previous data analyses, the authors implement several different statistical calculations. I think that it would be easier for the reader to follow the rationale for each calculation, if the authors would add a sentence or two, describing the considerations for each calculation method, such as chi-square analysis, one- or two-way ANOVA, Tukey’s test, etc.

*A short description is now included on why the chosen statistical tests were used.*

L192 - Figure S3: I assume that the authors calculate the statistical significance of the difference between incidence rate in temperatures below and above 10C. If they did, it would be more informative to show it. If not, I assume that it would be easy to calculate and show.

*Additional analysis was performed to compare temperature as a categorical variable differentiating above and below 10C. This is included in supplemental figure S3B. We included additional text regarding the results of a statistical test looking at above and below the 10C threshold. We identified no statistical significance in SARS-CoV-2 positivity rate for samples above vs below 10C (p=0.25).*

L232 - - what is Moran’s I? can the authors add a reference or an explanation?

*The Moran’s I statistic is now described in the body of the text with a reference.*

L266 – can the authors address the fact that only ~25% of the positive samples were “sequencable”, even after nested PCR? Was there any correlation between the initial Cq value and the ability to sequence the virus? Was it something else?

*We have added text in the discussion addressing the challenges of sequencing virus from these samples.*

*We include additional details and supplemental Fig S6 outlining sequencing results based on Ct value.*

L274 figure 4 – why was the word “in” added to each panel numbering in the legend? In panel D, was it WTD sequences? Human? Both? It is not explicitly stated.

*The figure legend has been edited for readability and clarity.*

L284-294 – this description includes technical details that I think should be included in the methods section, rather than in the results. The authors should consider revising it to be more concise from the technical aspect.

*The indicated text was moved from the results to the methods section.*

L311 Figure 5 – it is very difficult for me to understand all the symbols in the heat map, such as the different colors assigned for different genes (structural vs. non-structural?), the black short lines and red short line within the heat map, half way below the genome map, etc. please explain clearly what does each sign and symbol represent.

*A legend was added to further clarify the different colors used in the heatmap. This includes describing the gene as either structural or nonstructural and for the colors used in the heatmap itself.*

L346 Figure S6 – add “-“ in “time resolved”. Are the analyses based on complete genome sequencing? Please mention that in the figure legend or in the main text, when referring to the data presented in this figure.

*We have corrected the text to “time-resolved” trees (in what is now figure S8, old S6). The figure legend is updated to specify that only whole-genome sequences were used to generate these trees.*

L374 figure S7 – please include a short explanation on the dN/dS analysis, and explain in more details the figure – what does the heat scale (-1 to 1) represent? Are the numbers at the bottom representing individual sequences? Something else? The figure contains a lot of data that need to be more clearly presented to the reader.

*The figure legend has been updated to include more detail as requested. This includes describing the “VSP” of each individual sample described as the labels with numbers in the row called “all coding region” described as a summary of all coding regions for that white-tailed deer. The figure legend was updated to better describe the heat map scale. The body of the text includes an expanded description of the dN/dS analysis. This is now main figure 6.*

L389-407 figure 6 – where is the reference to panels 6E, 6F and 6G? as mentioned in the general comment regarding the null hypothesis that drives each experiment, what did the authors expect to find, when analyzing different tissues from the RPLN? It is likely that the viral load will vary between different parts, even in the same organ, and it is almost certain that it will vary between different animals. So what was the hypothesis here? Additionally, the minor differences in the sequences of VSP30917 tissues (panel 6E, 6F) warrant further explanation, from my experience, it is highly unlikely to find genetic differences of SARS-COV-2 genes within the same organ at a single time point. When e 550bp sequence is examined, this is even more exceptional. I think that the authors should address that either here or in the discussion section. I did not understand what does the short line (rod shape) represent in panel 6G? is it the average of the duplicate? Something else? Please explain.

*Figure 6 is now figure S7 in this resubmission. Reference to panels S7E, S7F, and S7G can be found in the body of the text “Not all sites produced amplicons, but those that did clustered tightly with other sequences from the same samples (Fig S7E, S7F).” and “To test whether biopsy samples from the core of lymph nodes showed different results than samples from superficial tissue trimming, we collected 8 WTD samples known to be SARS-CoV-2 positive and performed RT-qPCR in replicates for the core versus surface cuts (Fig S7G).”*

*In the revised draft, hypotheses, null hypothesis, and expected results have been added or expanded on for the RPLN tissue analysis.*

*We included additional details on the minor differences observed in VSP30917.*

Discussion section – this study includes a substantial amount of data that is new and important. It raises several important questions that I think should be addressed, at least in short, in the discussion.

First, the possible or empirically effect of SARS-CoV-2 infection on WTD was not mentioned. Are there any data on the clinical effect? Are the animals resistant to the virus? How long does the virus circulate in an individual WTD? Is it enough to accumulate mutations? These questions, in my opinion, are crucial for further understanding of the WTD population as a potential reservoir and source of SARS-CoV-2 spillback.

*We added details to address (1) clinical effects in WTD, (2) duration in individuals, and (3) previous work on accumulation of mutations within WTD. We thought it was best to include this in the introduction rather than the discussion section.*

In this context, are there any hypotheses regarding the transmission from humans to WTD? The virus is not known to survive long periods of time outside the host, and is not known to travel long distances away from an infected person. So the animals need to be in close proximity to infected individuals, in order to be infected. Is there any information in the literature regarding such possible transmission routes?

*In the revised discussion section, we review potential mechanisms of human-to-animal transmission.*

Another question that comes to mind when reading this study pertains to the serological status of the animals. Is there any information in the literature regarding WTD anti-SARS-CoV-2 antibodies? A survey that will include blood sampling and subsequent serological tests, such as ELISA and neutralization assays, could add important information on exposure of WTD populations to SARS-CoV-2.

*The revised discussion section now includes previous work on white-tailed deer SARS-CoV-2 serology. We did not have paired serum samples for the collected WTD to accurately assess anti-SARS-CoV-2 antibodies.*

I think that the authors should highlight several important points that are derived from the study, and were not emphasized in the discussion. These include the importance of WTD and possibly other animals as both sentinels and reservoir for SARS-CoV-2 and other pathogens, possible routes of spillover transmission that are not yet determined, and the potential of emergence and re-emergence of old and new variants, as a result of non-human circulating. These points could have a major effect on public health policies and should be addressed, at least in short.

*We have expanded the discussion section to further discuss WTD as a reservoir and sentinel species, routes of spillover transmission, emergence of new variance, and reemergence of ancestral variants.*

Lastly, it was somewhat unusual to sample lymph nodes and blood for SARS-CoV-2. I understand that the availability of the samples dictated this methodology, but I think that since this is not the common practice for detecting SARS-CoV-2 in humans, the authors should also address this, explaining the pros and cons of this sampling methodology, and mentioning the limitations of blood as a sampling material for SARS-CoV-2 viral load assessment. In this respect, if blood samples are available in a condition that allows serum separation and antibody testing, the above comment regarding serology study could be a starting point for a future study.

*We expanded the discussion section to include comments regarding white-tailed deer sample type and potential implications compared to what circulates in humans.*

L532 – can the authors explain why each sample was tested in duplicates? What were the Cq values obtained? Infected animals in acute state should have a low Cq value reflecting high viral load. If the smaples needed to be tested twice for each reaction – that means four different reactions - wouldn't that be excessively unnecessary?

*Samples were tested in duplicate for increased rigor. The animals tested in this project have unknown dates of initial infection, thus the expected Ct values could be highly variable depending on time since initial onset. By testing duplicates of two targets we expect to minimize noise and increase our sensitivity to detect infections even if they occur at low viral loads. These duplicates also help protect against false positives. We were not able to test whether the animals were acutely infected since sampling only occurred after death.*

L576 – The entire explanation regarding the Spike-targeted Amplicon Sequencing is lacking details – which regions were amplified? which primer sets were used? What was the first reaction targeting? Within that region, what was targeted in the nested reaction? Were any variant-specific mutations targeted in this procedure? If so, which ones? Please elaborate on the design of the analysis.

*The design of the analysis is now described in more detail in the revised paper, including which specific regions in the viral genome were targeted by primers.*

L577 – I could not find any primer sequences in table S2. Please provide the table with the primer details.

*Primer information outlined at the end of Supplemental S4 (now S6) was moved to a dedicated Supplemental file (new S3) to make it easier to locate. We now spell this out in the methods.*

L612-616 – please add references as links to the reagents supplier relevant website.

*Supplemental table S4 (now S6) has been modified as requested to include links to the relevant reagents used in the assays.*

Again we thank the reviewers and editor for their helpful comments. We hope the paper is now suitable for publication in PLOS Pathogens.

Sincerely,

Rick Bushman
