# Supplementary material for: Evolution of SARS-CoV-2 in white-tailed deer in Pennsylvania 2021–2024
Source: PLoS Pathog. 2025 Jan 24;21(1):e1012883. doi: 10.1371/journal.ppat.1012883 (PMC11781694; doi:10.1371/journal.ppat.1012883)
Supplement: Attachment — Submitted filename: MarquesPLOSPathRespv14.docx [file ppat.1012883.s013.docx]

**Department of Microbiology**	Frederic Bushman, Ph.D.

3610 Hamilton Walk	Professor and Chair

426A Johnson Pavilion

Philadelphia, PA 19104-6076

Tel 215.573.8732 Fax 215.573.4856

[bushman@mail.med.upenn.edu](mailto:bushman@mail.med.upenn.edu)

www.med.upenn.edu

January 2, 2025

Re: Response to reviewers’ comments on Manuscript PPATHOGENS-D-24-01513 entitled "Evolution of SARS-CoV-2 in White-tailed Deer in Pennsylvania 2021-2024".

Dear Madam or Sir,

Thanks very much to the reviewers for their helpful comments on our paper PPATHOGENS-D-24-01513 entitled "Evolution of SARS-CoV-2 in White-tailed Deer in Pennsylvania 2021-2024". We have edited the manuscript as suggested. Below we list our responses to reviewers in italics beneath each comment.

**General comments**

The authors did, as they mention in the revision note, made many improvements in the manuscript, but there are still many issues that need to be addressed, as I detail in the specific comments below. A major issue in my opinion, is the amount of data that is included in the manuscript. The authors performed a very comprehensive study. However, including a total of 14 figures and 11-12 tables and ~80 referenced citations, is a very large amount of data for a single publication. In several instances, the authors perform very extensive analyses to show the absence or presence of associations or correlations between different variables, in a manner that in my opinion is sometimes overdoing. For example, a simple graph or table is sufficient to show that WTD sex had no association with SARS-COV-2 infection rate. Further calculations and statistical tests are in some cases unnecessary and just add unnecessary information that extends the length of the manuscript.

*We thank the reviewer for their thorough comments. In response, we have reduced the number of figures and tables. There are now 11 figures including 6 main figures and 5 supplemental figures. There are now 4 tables including 1 table in the manuscript and 3 supplemental tables. We believe that reducing any further would detract from the conclusions reached in the study.*

*The reviewer suggests that some conclusions should be drawn based on figures and text without statistical tests. We believe that performing the statistical tests as written in the manuscript are required to maintain scientific rigor; however, in the spirit of the reviewer’s comments, we have simplified the presentation.*

It is up to the Editors, of course, to decide what the allowed manuscript length is. I think that shortening the current version might render it more readable for the audience and improve its impact.

*We shortened the manuscript by 573 words.*

Another issue to consider is the accuracy of the tables labeling. Within the current version, there are several places where the referencing is incorrect or inconsistent.

*We have corrected call outs to the tables.*

As I noted for the first version of the manuscript, some of the figures and their legends are very complex, contain a lot of data, and are sometimes difficult to understand. I made specific comments that pertain to these cases. I think that it would be beneficial to re-examine all the figures and their legends and simplify them when relevant.

*Legends were simplified to contain just the most important information. Some figures were removed.*

I did not understand why the supplementary tables legends are placed in-line with the main text. Shouldn’t they be place in the supplementary files?

*Supplemental Information has been described at the end of the manuscript.*

**Specific comments**

L75 – Any reference for the statement?

*The text has been revised to clarify references, and a reference added at the indicated line.*

L77 - it is up to the Editor to decide, but i am not sure that 20 references are needed to make the point that animal species are infected with SARS-COV-2

*We checked the literature for redundancy. All citations are required to support the claim that at least 55 species are susceptible.*

L84 - I think that the legend should be placed with the table in the supp. material. Is there a particular reason that it appears here in the main text?

*Legend placement is as dictated by the journal guidelines.*

L119 – any reference for the statement?

*This statement is made as a concluding statement summarizing the cited claims established earlier in the paragraph.*

Figure 2 (L249) – panel (A) I am not sure I understood what the authors mean by “counts” – is it the total number of examined animals in a given time point? Is it the total number of samples tested in a given time point? Is it something else? The statistical significance refers to the difference between the percentages of positive samples in each season? It is not clear what does the statistical significance refer to. In panel D, the highest calculated average positivity was in January 2024, when SARS-COV-2 in human was declining, compared to the Previous 2-3 years. Do the authors related to that in the discussion? Is there a possible explanation for what seems like an increased in positivity in WTD, when there is a decrease in prevalence among humans?

*The text has been updated for clarity. A single count refers to a single white-tailed deer. Statistical tests were performed using the counts of positive verses negative white-tailed deer. This is clarified in the revised text.*

*We do not have accurate data for positivity rate in humans during the course of this study. For scientific rigor, we would need data local to the area where sampling occurring in WTD for the same time periods without a change in how testing in humans were done. This dataset is not available to our knowledge therefore a rigorous comparison cannot be made.*

L311 – perhaps “higher than” or “above” would be more appropriate than “more than”.

*The text was revised as recommended.*

Figure S2 – the X and Y axes are not labeled, making less clear to the reader.

*Figure S2 was removed to reduce the number of figures and shorten the manuscript length.*

Figure S3 – I think it might be clearer to denote the percentage of positive vs. negative samples for each sampling group, rather than presenting it as bar graph portion.

*The data is presented in the text as a percentage of positive vs negative samples for each sampling group. The bar graph in S3 panel B was removed.*

Figures S4+S5 – it might be better to join the two figure into one multi-panel figure, to reduce the amount of supplementary items without loosing information.

*Figure S4 was removed to reduce the number of figures and shorten the paper length.*

Figure S6 – there are several aspects that are not clear to me regarding the calculation of sequence success probability: what is the amplified sequence length? It is not specifically noted. it looks like the real-time PCR is not very sensitive; Previous experience in our lab suggests that 51% success for sequences derived from Ct of 23-28 is rather low. I would expect a much higher success rate, closer to 95%. Similarly, probability of 12% sequencing for samples with Ct value of 40 seems very high. We usually do not succeed in amplifying “sequenceable” reads from samples with such values. I think that these predictions should be at least briefly addressed in the discussion. Lastly, as I mentioned for previous figures, it was not clear to me why the term “count” is used, instead of “samples” or “individuals”. It might be a common term in that respect but here it is somewhat confusing.

*Figure S6 was removed to reduce the number of figures and shorten the manuscript length. Success rate of sequencing is dependent on more than Ct value alone. RT-qPCR target regions are significantly smaller than sequencing regions, therefore degradation of RNA is likely to reduce the success rate of sequencing. Our previously publications using human samples (like saliva and NP swabs) have a much higher success rate of sequencing for the Ct values as the reviewer describes. However, collection occurred in clinical settings and maintained ideal cold chain storage leading up to testing. As described in this manuscript, the sample collection process was not ideal for preserving RNA since samples were originally collected and handled for prion testing. As described in the manuscript, the sequencing process used for this metric was described immediately before Figure S6 was mentioned (line 377 to 381 of the second submission). This is specified in more detail in the methods section as being a 550 base pair region. We revised the text to include further discussion of these technical specifics.*

L394 – I did not understand this sentence. The table (S3) lists the accession numbers of sequences used for the analysis. Where are the primers listed?

*We removed the mention of Table S3. The table of primers was removed to reduce the number of supplemental materials – this information was redundant since the primers are listed in the manuscript’s methods section.*

L402-417 - Figure 4. I did not understand why the file size of the figure is over 80Mb. It makes it a little bit annoying to have to download such a large file, which does not include microscope images or other photographs, only illustrations. I understand the need to create a sharp image of the map, but still, 81Mb seems too much. I think that it would be easier for readers to download high resolution images in a smaller size.

*Figure 4 has been compressed to be a smaller file size.*

L419-420 – the term “quantified Ct values” should be used when Ct values are converted to target nucleic acid concentration, in this case, SARS-CoV-2 genome copies. This is not what is presented in figure S7. So, I think the word “quantified’ should be replaced with “determined” or “established”.

*The text has been revised as suggested.*

L427-441 Figure S7 - the authors performed several statistical analyses that showed, eventually, that there was a statistically-significant difference between different animals, but not within RPLN sites of the same animal. From my experience, this is pretty much obvious that different individuals (animals or humans) will have different viral loads. I do not understand why the statistical analysis was necessary (to support or reject a null hypothesis?).

*We believe that this analysis is important because it tests the hypothesis if different anatomic locations within a lymph node have an impact on Ct value while controlling for variability among animals. The model, albeit simple, performs well and captures the results.*

L454-456 (Figure S7) – it is not specified what was the analyzed part of the viral genome – the entire spike gene? The RBD coding sequence? The authors need to sate or refer to the amplicon positions in the viral genome. For example: region between position 21,400 and 22,400, in reference sequence Genbank no. Otherwise it is impossible to fully appreciate the analysis.

*The manuscript was revised to include the requested specifics.*

L458 – 1. in the beginning of the sentence, should be written CT VALUES or VIRAL LOAD comparison….. 2. I am not sure that the sentence “x-axis shows the lymph node sampled” is correct. In the figure that I downloaded, The X-axis shows the numbers of the sampled animals. 3. There is no explanation in the figure legend, what do the bars represent.

*The line in question references the legend for panel G. While sequencing was performed on three animals, the RT-qPCR analysis was performed on 8 animals. There are 8 columns along the x axis with each column representing a lymph node sampled from a single animal. New text has been added to describe panel G.*

L466-47 – if the regions at the end of the sequenced amplicon are sequenced at low quality, they can be trimmed to leave only the segments for which the base calling is with high degree of certainty. The authors may take that into consideration, either when performing the analysis, or when discussing the results.

*We performed trimming based on length from the edges of the reads. It would not be fair to exclude a larger region for just a subset of samples to make the data appear cleaner. As described in the manuscript methods, this analysis used sanger data, thus quality score metrics are not available for individual base positions. A set trimming length was uniformly used across all samples and data presented as it was analyzed.*

L482 – A Ct difference of 1.7 may be a result of run-to-run variation. In order to establish that this indeed results from the cut depth, I think that a larger sample size needs to be tested. Therefore, I suggest that the authors make the statement with caution, regarding the association between cut depth and Ct values, when based upon the described dataset.

*The samples were run with replicates; the manuscript is now revised to make this point more clearly. The Ct increase of 1.7 represents an approximately 3.2-fold increase in viral RNA copies (2^1.7^). We believe that in our analysis we appropriately controlled for sample-to-sample variation to draw conclusions based on the depth of the biopsy cut. The manuscript has now been edited to include more details on this analysis.*

Figure 5 – same comment as for figure 4 regarding excessive file size.

*The file size has been reduced as requested.*

L494 – Newly determined or newly sequenced WTD samples? If not the viral sequence, what was determined?

*The text was edited for clarity as suggested.*

L497 – Perhaps “beginning” would be more appropriate than “Start”.

*The text was edited for clarity as suggested.*

L501 – the title for figure 5 is misleading – the Phylodynamics are of SARS-CoV-2 genomes collected from WTD, not of WTD genomes….

Similarly, in the legend itself, this should be corrected accordingly.

*Edits were made for clarity as suggested.*

L513-514 – the word “the” appears twice. I did not understand what does the “root” sequence represent. If these are the sequences that the tree is rooted by, shouldn’t the tree architecture be different? There is no referring to the root sequences in the text, so this is somewhat confusing. There is no referring in the text to the differences between structural and non-structural genes, so the color-coding in panel B (Figure 5) is overloaded with information that does not add anything to the reader’s understanding.

I could not download table S4 of the revised version. The old version table S4 is of reagents.

*The text has been edited for clarity, including more clearly defining how root sequences were selected. The figure was edited to remove colors of structural vs non-structural coding regions.*

L574 - Fig S8 – again, the fact that the genomes are of SARS-CoV-2 is not reflected in the title.

*The text was edited for clarity.*

L584 – HPD is a term that is used frequently in this part, so I think the authors should briefly explain what it stands for, at least in the first place where they use it in the manuscript.

*The highest posterior density is a term used to describe the type of Bayesian maximum credibility phylogenetic analysis used in this work and the most common method for determining time-resolved phylodynamics. It represents the region of posterior probability distribution which is the smallest possible interval that contains a given probability mass under the posterior distribution. This selection set at 95% for the distribution ensures that the reported range of values are among the most probable to occur. In other words, it is like picking the most “crowded” part of a probability distribution. Imagine you have a bunch of possible answers to a question, each with some likelihood. To find a range that is 95% of all the likelihood, the HPD would be the smallest set of answers where the probabilities are stacked as densely as possible – thus choosing the most likely answers first until you’ve captured that 95%.*

*We edit the text to include a brief description of this which is included in the related methods section.*

L596 – Fig6 – as for previous figures, this figure contains a lot of information that is not completely clearly explained. In panel A, I could not find an explanation for the difference between circle or diamond data points. I also think that using so many colors to differentiate between the different genes is unnecessary complicating the interpretation. I think that a schematic map of the genome adjacent to the X-axis would be much easier to follow than all the colors.

*The figure legend was revised for clarity. The coding region coloring was simplified to have fewer colors and colors only for coding regions that were mentioned in the text.*

L609-611 – this description is confusion. I think a simple formula, either in the text or in the figure, would be much easier for the reader to follow.

*A formula was included in the manuscript to aid in clarity.*

L624 - Poisson regression ANALYSIS

*The text was edited for clarity.*

L629-640 – this is not a description of the results, but rather an interpretation of them. It should therefore be mentioned (briefly) in the discussion, not in the results section.

*This text was modified and moved to the discussion section.*

L649-651 – isn’t the substitution going from Wuhan strain to XBB? The text says the opposite. If I understood it, it says that there were substitutions from the XBB to the Wuhan, and not the opposite. I suggest that the authors would consider rephrasing this sentence to make it clearer. If this I a reversion, it was not clearly described.

*The text was edited for clarity.*

L732 – Should be “evolution rates of SARS-COV-2 in WTD”

*The text was edited for clarity.*

L744-751 – I think that there was not enough information (sampling size too small) to conclude anything definite regarding the association or absence of association between SC-2 and CWD. In the study’s setting, there was no association.

We have retained the analysis because it achieves statistical significance, but in the spirit of the reviewer’s comment we have mentioned that small sample size is a limitation on our conclusion.

L771 – should be “some of the statistical ANALYSES”, not “analysis”.

*The text was edited for clarity.*

L686 – I did not understand which cause of death was calculated, and what did it have to do with the description of how the temperature values were determined for the study region.

*From our earlier analysis, cause of death was the only demographic feature that had statistically significant associations with SARS-CoV-2 positivity which warranted their inclusion in the Bayesian model of temperature. The cause of death type “target removal” were found to be significantly more likely to be SARS-CoV-2 positive and these typically occurred in narrow windows of time with clusters of deer so not accounting for this confounding variable would be an oversight. We deem this model to be the most rigorous and accurate. We have edited the presentation to make this point more clearly.*

L882- the title and content of Supp. Table 3 that I downloaded are “Samples and accession numbers for sequence data used in this study”. So either I downloaded the wrong table or there is a confusion in the supplementary tables.

*Supplemental tables have been reduced and renamed. They should now match the call outs in the text, or the call outs have been removed.*

L927 – The Supp Table S5 that I downloaded, contains information on mutations and how many times they occur. This is the same S5 table that is referenced in the results section (L646 and other places). The authors should correct the numbering of all tables and make sure all tables and figures are accurately numbered and referenced in the text.

*Supplemental tables have been reduced and renamed. They should now match the call outs in the text or the call outs have been removed.*

L940 – as mentioned above, Table S3 that is mentioned here is not the same as the table that is reference in line 882. Again, the authors need to correct the tables numbering across the manuscript.

*Supplemental tables have been reduced and renamed. They should now match the call outs in the text or the call outs have been removed.*

L950 – what is ggtree?

*The software ggtree is an R package for visualizing phylogenetic trees. The text has been revised to specify this.*

Again we thank the reviewers and editor for their helpful comments. We hope the paper is now suitable for publication in PLOS Pathogens.

Sincerely,

Rick Bushman
